# The anti-inflammatory efficacy of melanocortin drugs is influenced by genetic variation at *MC1R*

**DOI:** 10.1038/s41598-026-57399-0

**Published:** 2026-06-11

**Authors:** Natalya Khodeneva, Camilla S. A. Davan-Wetton, Thomas E. N. Jonassen, Mauro Perretti, Trinidad Montero-Melendez

**Affiliations:** 1 The William Harvey Research Institute, Queen Mary University of London, Charterhouse Square, London, EC1M 6BQ UK; 2 Department of Biomedical Sciences, University of Copenhagen, Copenhagen, DK-2200 Denmark; 3 SynAct Pharma ApS, Holte, 2840 Denmark; 4 Centre for Inflammation and Therapeutic Innovation, Queen Mary University of London, Charterhouse Square, London, EC1M 6BQ UK

**Keywords:** Melanocortin, Pharmacogenetics, Precision medicine, Drug development, Genetic diversity, Computational biology and bioinformatics, Diseases, Drug discovery, Genetics, Immunology, Medical research

## Abstract

The melanocortin 1 receptor (*MC1R*) is a pro-resolving anti-inflammatory target under clinical development for scleroderma, arthritis, light intolerance or melanoma prevention. Genetic diversity at *MC1R* is high in certain populations, with some variants associated with loss-of function (LoF) resulting in red hair and poor tanning response. However, how these variants influence the anti-inflammatory efficacy of drug candidates targeting *MC1R* is unknown. We analysed the impact of 30 variants on receptor signalling (cAMP and phospho-ERK) and the anti-inflammatory response to nine agonists on the whole-blood assay on healthy volunteers. LoF presents as a continuum rather than as a binary characteristic (benign/pathogenic) and differentially affects each signalling pathway, undermining the usefulness of in silico tools to predict variants pathogenicity. Moreover, variants affected compounds differently, even causing LoF, no effect or gain-of-function depending on the compound tested. We identified responders and non-responders to melanocortin compounds, and the efficacy of most compounds (determined as reduction of cytokine release) was diminished by the presence of variants at *MC1R*. Carrying red-hair variants (RHC) also associated with reduced efficacy but not having light skin phototype. These data encourage the incorporation of pharmacogenetics strategies during melanocortin drug development programs to ensure targeted interventions for maximal efficacy and safety.

**Supplementary Information:**

The online version contains supplementary material available at 10.1038/s41598-026-57399-0.

## Introduction

Providing patients with the right medication based on their genetic make-up is the ultimate promise of pharmacogenetics^1^. By studying how the variability on genes influences the therapeutic benefit of a drug, can positively impact all stakeholders of the process of drug development, i.e. patients, industry and national health services. Patients will not receive a drug that does not work while only risking side effects. The chances of success of clinical trials increase, as non-responders may be identified early, guiding participant recruitment. Furthermore, it was estimated that the economic burden to the United Kingdon National Health Service due to prescription of ineffective drugs could be as high as >£500 M annually^2^.

G-protein coupled receptors (GPCRs) are the largest class of targets for approved drugs (~ 34%). However, genetic variation on these genes is also vast: on average, each individual carries 68 missense variations in the coding region on more than 30 GPCR drug targets and, on average, 8 of these variants are already known to be associated with altered drug response^2^. There is also evidence that therapeutic targets with supporting genetic information are more likely to succeed at clinical trials and achieve regulatory approval^3–5^. Remarkably, despite this knowledge, pharmacogenetics approaches are seldom incorporated into R&D programmes, neither at pre-clinical nor clinical stages.

The gene encoding for the melanocortin 1 receptor (*MC1R*), a class A GPCR, represents an excellent opportunity for the application of pharmacogenetics. Numerous therapies targeting this receptor are being developed^6^ at clinical stage, including dersimelagon (Mitsubishi Tanabe Pharma) for scleroderma, afamelanotide (Clinuvel Pharmaceuticals) for skin disorders, PL8177 (Palatin Technologies) for ulcerative colitis, or resomelagon (SynAct Pharma) for rheumatoid arthritis. However, in spite of the substantial genetic diversity observed for *MC1R*, which indeed affects its activity, pharmacogenetics is not integrated in current development programmes.

Besides its therapeutic potential as an anti-inflammatory target due to its expression in immune cells and other cells like fibroblasts^6,7^, this receptor is well known for its prominent role in melanocytes, influencing skin and hair pigmentation. Activation of melanocortin receptor type 1 (MC_1_)on melanocytes by its endogenous ligand alpha melanocyte-stimulating hormone (⍺MSH) induces the pigmentary machinery leading to the production of eumelanin (brown/black). The default phenotype in the absence of MC_1_ activation is the generation of pheomelanin (yellow/red)^8,9^. Then, the phenotypes most strongly associated with certain *MC1R* variants are red hair, freckles and the response to ultraviolet (UV) radiation exposure (i.e. tanning)^10,11^. Given that UV radiation has been one of the major selective pressures driving human evolution, it is not unexpected that a pigmentary gene like *MC1R* has been subjected to alterations throughout time and geographical locations since our ancestors left Africa ~ 60,000 years ago, resulting in a wide range of variants that may exert varying levels of influence on receptor function.

In this study we address how diversity at *MC1R* gene may influence the efficacy of melanocortin compounds. Specifically, we aimed to understand, first, if carriers of loss-of-function (LoF) variants at *MC1R* would respond to a melanocortin therapy, and second, whether easily accessible information like in silico predictors of pathogenicity at SNPs level, or patients’ skin phototype, could predict drug responsiveness.

## Results

### Analysis of MC1R gene diversity in humans and selection of variants

**Fig. 1:**
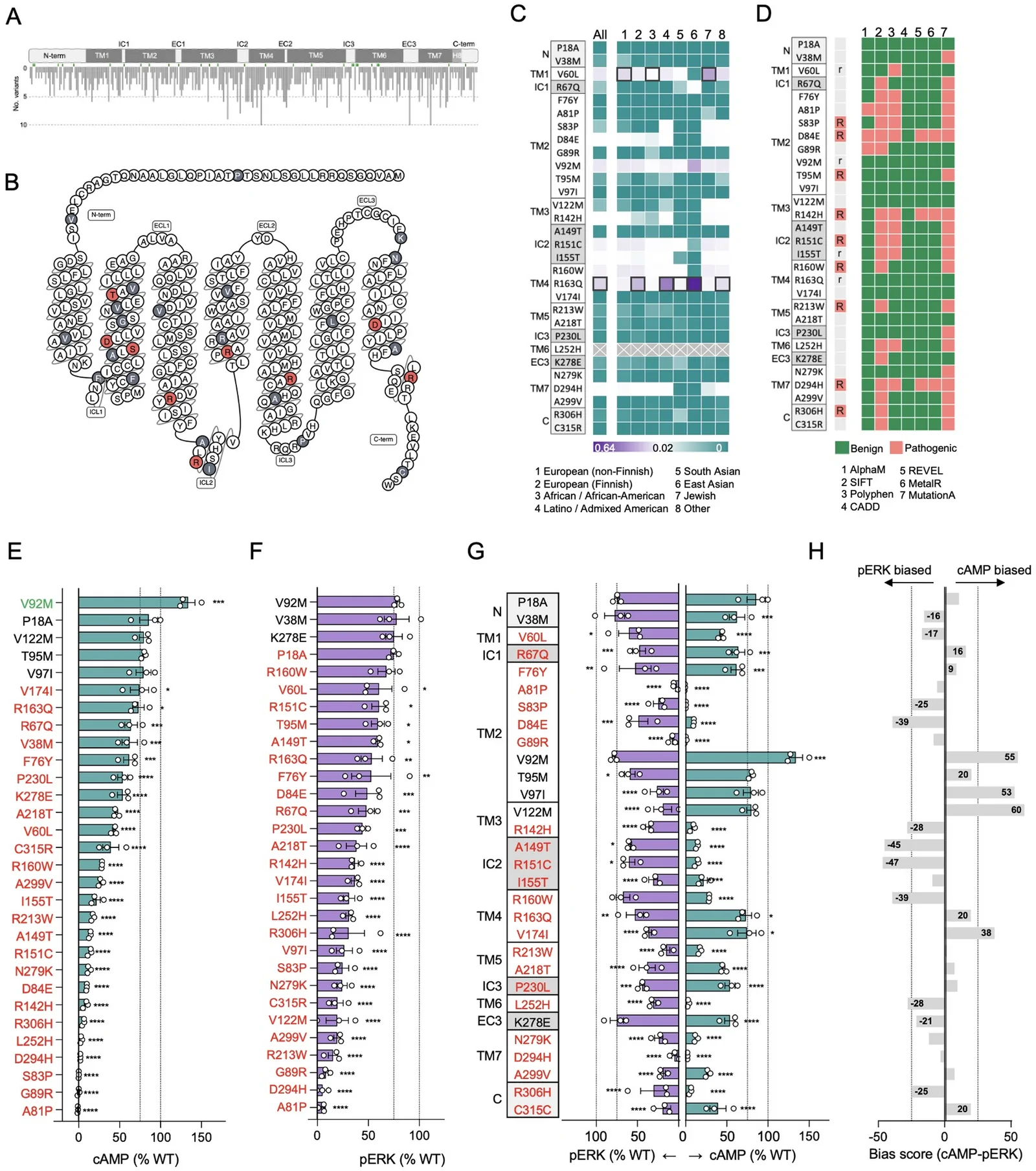
Genetic variation at *MC1R* and signalling responses to ⍺MSH. (**A**) Distribution of variants along the protein structure of *MC1R* obtained from Ensembl (Ref ENSG00000258839; GRCh38.p13). Gray bars indicate the number of variants in a given position. Green ticks indicate amino acid positions with no variants reported. (**B**) Snake plot representing the 30 variants selected in this study (red= evidence of association with red hair phenotype). (**C**) Allele frequency of the 30 selected variants obtained from gnomAD v2.1.1 -high frequency (purple) or low frequency (teal). Black boxes indicate the most frequent variant in each population. (**D**) Pathogenicity predictions obtained from AlphaMissense and Ensembl. Green and red colour indicate whether the predictor identified the variant as benign or pathogenic respectively. Weak (r) or strong (R) association with red hair phenotype, as reported in Morgan et al.^*12*^. (**E**,**F**) cAMP and pERK responses in HEK293 cells transfected with *MC1R* variants upon stimulation with 10µM ⍺MSH for 15 and 5 min respectively. Data is presented as percentage of response obtained in cells transfected with the wild type (WT) form of *MC1R*. Values ≥ 25% or ≤ 25% vs. wild type were considered GoF (in green) or LoF (in red) respectively. (**E**) cAMP responses ranked by response. (**F**) pERK responses ranked by response. (**G**) cAMP and pERK responses ranked by amino acid order. (**H**) Biased score calculated by subtracting pERK from cAMP responses. Values near zero indicate lack of bias. Data represent mean ± SEM of *n* = 3 and analysed by one-way ANOVA with Dunnett’s multiple comparison test vs. WT (E-G); *p < 0.05, **p < 0.01, ***p < 0.001, ****p < 0.0001.

To obtain a complete overview of the genetic diversity at *MC1R*, we extracted the data from Ensembl and Genome Aggregation Database (gnomAD). We identified a total of 900 variants distributed throughout the entire sequence of the protein (Fig. 1A), including 433 missense variants. The highest global allele frequency was found for silent T314T and missense R163Q variants (Fig. S1A). Homozygosity was rare and only detected on 37 of the 900 variants (Fig. S1B). We selected 30 variants (Figs. 1B, S2), including V60L (most common in European, African/American and Jewish populations), R163Q (highest frequencies in Finnish, Latino, and Asian populations), and others like D84E or D294H, commonly associated with red hair colour (referred to as ‘RHC’ variants), suggesting strong loss of function (Fig. 1C). Predicted pathogenicity for them was obtained from Ensembl and AlphaMissense and compared to reported association with red hair^12^ as an indicator of pathogenicity (Fig. 1D). For some variants (D84E, R142H, D294H), most predictors accurately identified pathogenicity. However, other reported loss-of-function (LoF) variants (T95M, R160W) were wrongly classified as benign, indicating the need for empirical data to reliably determine LoF of a variant.

### Detection of loss of function and signalling bias at MC1R

To analyse variants functionality, we measured the ability of alpha-melanocyte stimulating hormone (⍺MSH) to induce cAMP accumulation and phosphorylation of extracellular signal-regulated kinase (pERK), on HEK293 cells transfected with each of the 30 selected variants. Herein, we define LoF as reduction of at least 25% activity -i.e. ≤75% compared to wild type (WT) activity-, and GoF as increased activity of ≥ 25% -i.e. ≥125% of WT activity. On cAMP (Fig. 1E), LoF was observed for the majority of the variants (25), reaching in several cases (L252H, D294H, S83P, G89R or A81P) a complete abrogation of the activity. Only one variant (V92M) displayed gain of function. Similarly, most variants impaired the ability to induce pERK, while GoF was not observed for any of them (Fig. 1F). In line with these findings, cAMP (but not pERK) was significantly reduced in RHC variants, with the unexpected exception of T95M (Fig. S3A).

Ranking the data by amino acid position (Fig. 1G) revealed a strong dependency of a region at the transmembrane 2 domain (TM2, positions 81 to 89) to maintain the activity at both signalling pathways as well as TM7 (e.g. D294H) and C-terminus domains. However, other regions associated with differential effects, like variants in position 92–97 of TM2 causing signalling bias towards cAMP, while the region around intracellular domain 2 (IC2) presented a preference for pERK signalling (Fig. 1H).

Receptor expression could be found either homogeneously distributed through the cell, or intracellularly retained, possibly in the Golgi or endoplasmic reticulum (65.5% and 34.5% for WT *MC1R*) (Fig. S3B). Although the proportion of cells displaying each type of receptor distribution varies across variants, we did not find evidence that receptor expression pattern could explain differences observed in cAMP or pERK signalling (Fig. S3C, D).

On cAMP, we observed a strong significant correlation between intrinsic and ligand-dependent (⍺MSH) activity, while for pERK, this association was weaker (Fig. S4A-G). Overall, variants reducing intrinsic activity also reduced ligand-dependent activity. Exceptions include V92M, which reduced cAMP intrinsic activity but increased ⍺MSH activity, or variant S83P, with normal pERK intrinsic activity but reduced ⍺MSH activity. Significant correlation was observed between cAMP and pERK induced by ⍺MSH, while this association was not observed at the ligand-independent activity. This suggests that the intrinsic activity at pERK pathway is less susceptible to be disrupted by the variants, hence displaying a prominent bias towards pERK (Fig. S4E), while on the contrary, effective cAMP engagement seems to be more dependent on an intact protein.

### In silico predictors of pathogenicity fail to predict loss of function at MC1R

**Fig. 2:**
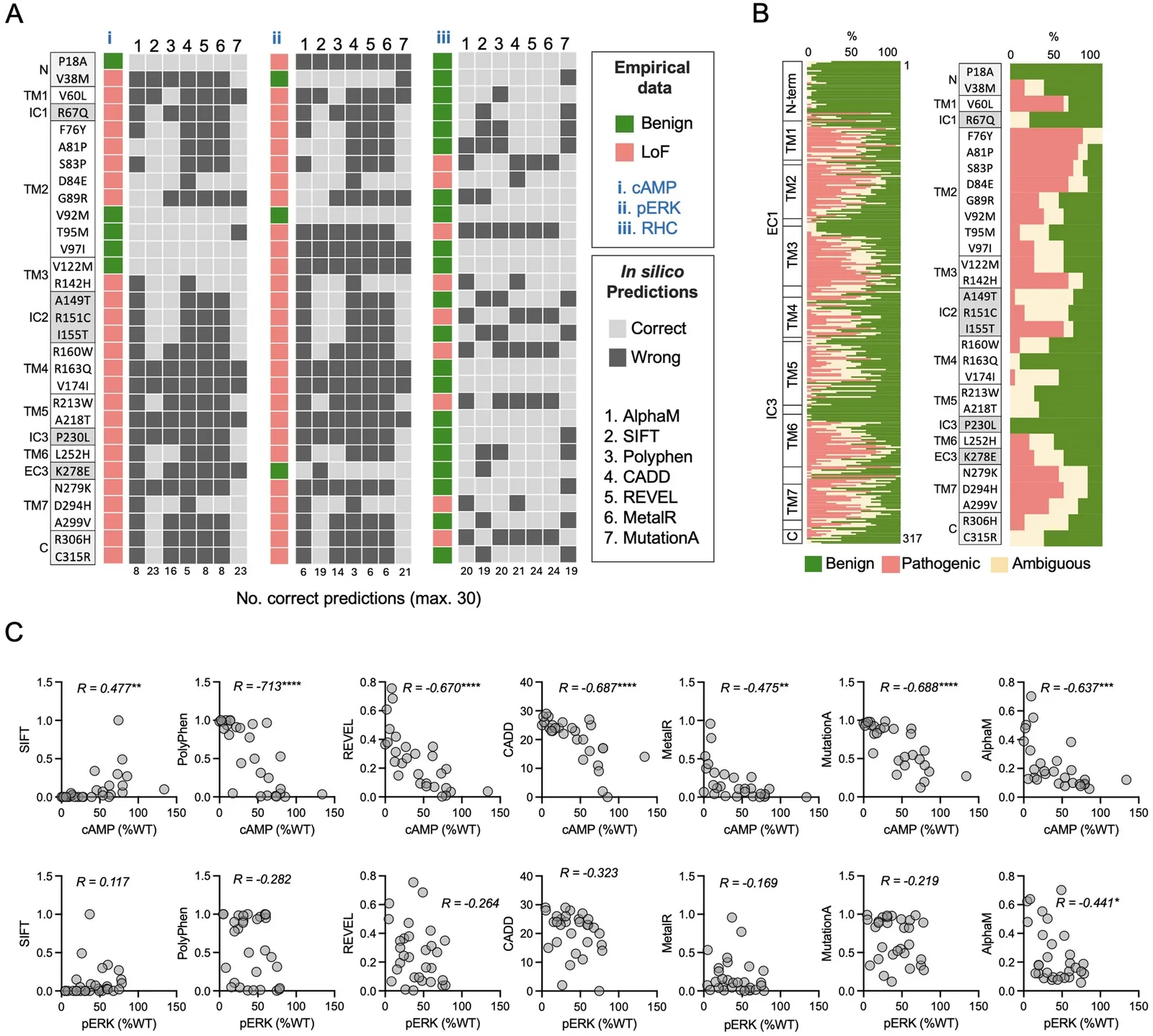
Comparison of pathogenicity predictions with empirical data. (**A**) Data on cAMP and pERK responses to ⍺MSH on HEK293 cells transfected with each of the *MC1R* variants from Fig. 1 are represented in columns i and ii respectively. Data on the association with red hair obtained from Morgan et al.^*12*^ are represented in column iii. Red colour indicates pathogenicity (i.e. LoF at cAMP or ERK, or positive association with red hair). The accuracy of the seven in silico predictors is shown in columns 1–7: correct predictions are displayed in light grey and incorrect predictions in dark grey. The total number of correct predictions (i.e. the sum of grey squares in each column) for each predictor tool is shown below the heat maps. (**B**) Predictions obtained with AlphaMissense machine-learning system for the entire *MC1R* amino acid sequence and for the 30 positions on this study. A prediction of pathogenicity was obtained for all possible single amino acid changes at each position (e.g. if a position with an alanine is changed with each of the remaining 19 possible amino acids). Data is represented as % of changes (i.e. 100% equals 19 changes). (**C**) Pearson correlation between the predictors scores and the cAMP and pERK responses by ⍺MSH (as % of wild type, average of *n* = 3); **p < 0.01, ***p < 0.001, ****p < 0.0001.

If in silico tools (e.g. Polyphen, AlphaMissense, etc.) could reliably predict LoF, this could easily be used to inform pharmacogenetics approaches during drug development. We used the extensive empirical information at each variant generated above to validate their usefulness. Overall, the number of correct predictions of LoF at cAMP and pERK was very low for most algorithms (Fig. 2A). Although the likelihood of a pathogenic prediction to be true (positive predictive values, PPV^13^ and specificity (ability to detect benign) were high for most predictors at cAMP and pERK (Fig. S5A, B), the sensitivity was very low due to the detection of many false negatives. Prediction of red hair was also generally poor (Fig. S5C). We also observed a general disagreement between predictors. Only the benign variant V92M was correctly predicted by all tools and for the three functional outcomes. Similar poor performance of in silico predictors was obtained for *MC4R* gene function, which signalling data for 41 genetic variants was reported by Lotta *et at*^14^ (Fig. S5D, E). Deeper insights can be derived from AlphaMissense^15^, from which we obtained predictions for each of the 20 possible amino acids that could exist at each position. Predicted pathogenicity was associated with transmembrane domains (Fig. 2B) and the opposite for the N-terminus, EC1 and IC3 domains. Certain positions appear to be highly susceptible, as any amino acid change would cause pathogenicity (e.g. F76) while others are predicted pathogenic only with certain amino acid changes (e.g. V38). Finally, when using the actual numeric score generated by the predictors rather than the classification as benign/pathogenic, the correlation was modest but significant, although only for cAMP (Fig. 2C), suggesting that refinement of scores interpretation may turn these tools more valuable.

In summary, the performance of existing predictors on *MC1R* variants is not satisfactory to inform drug development strategies or disease risk evaluation.

### MC1R variants differentially affect the signalling responses of melanocortin drugs

**Fig. 3:**
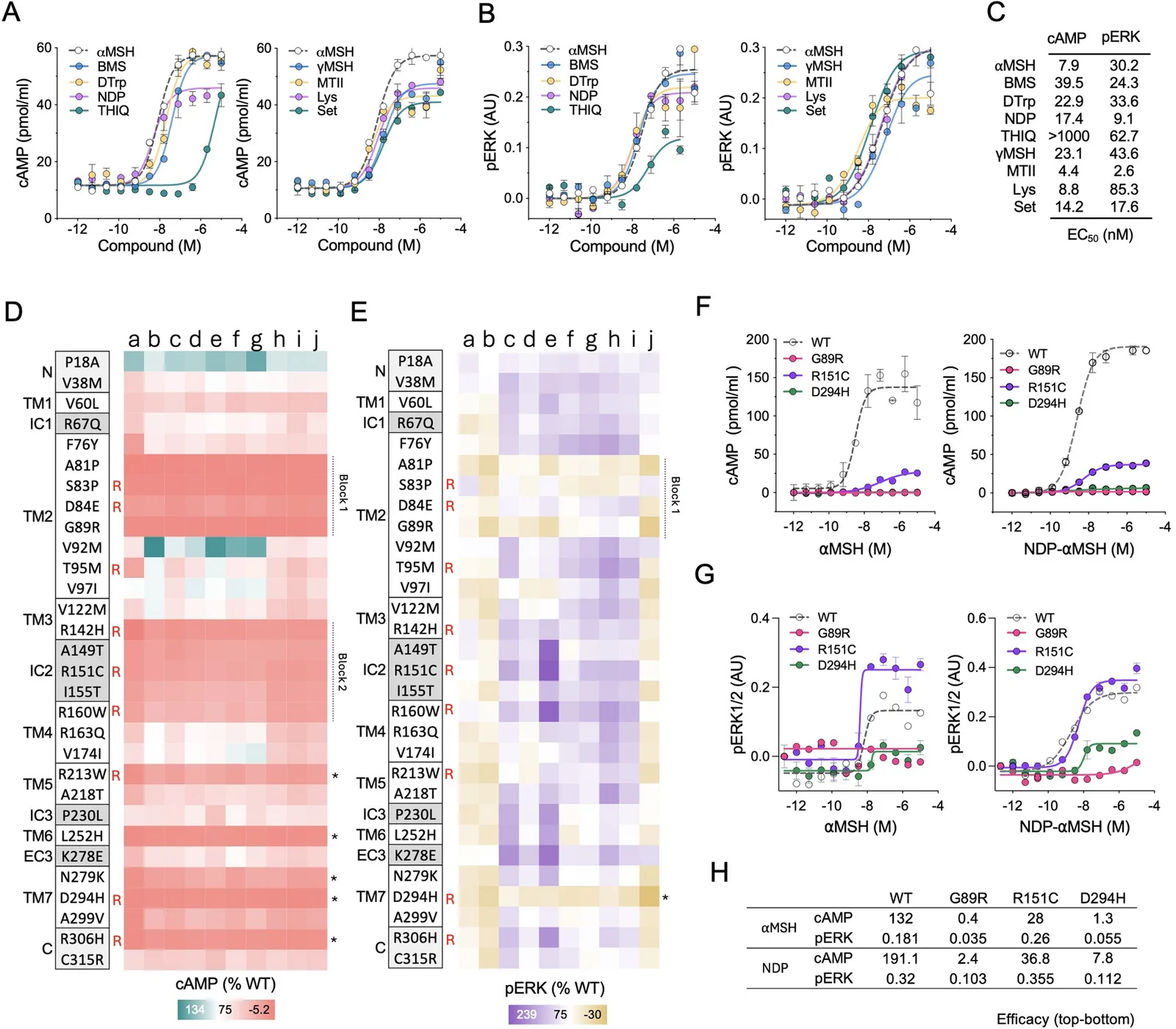
Effect of *MC1R* variants on the efficacy of melanocortin compounds at cAMP and pERK. (**A**–**C**) cAMP (**A**) and pERK (**B**) full concentration responses were measured in HEK293 cells transfected with wild type (WT) form of *MC1R* upon stimulation with melanocortin compounds for 15 and 5 min respectively. Potency values (EC_50_) (**C**) for all compounds analysed using log(agonist) vs. response - variable slope (four parameters model). (**D**,**E**) cAMP and pERK pathways in cells transfected with the *MC1R* variants upon stimulation with 10 µM melanocortin compounds (a: vehicle, b: ⍺MSH, c: BMS-470539, d: DTrp^8^-γMSH, e: NDP-⍺MSH, f: THIQ, g: γMSH, h: MTII, i: Lys-γ_3_MSH, j: setmelanotide) for 15 and 5 min respectively. Data is presented as % of WT response. Values ≥ 25% or ≤ 25% vs. WT were considered GoF or LoF respectively. Regions with strong and consistent LoF across compounds are labelled as Block 1, 2 or marked with asterisks. R indicates strong association with red hair (RHC variant). Colour scheme indicates high/low activity for cAMP (teal/red) and pERK (purple/yellow). (**F**–**H**) Full concentration-response curves by ⍺MSH and NDP-⍺MSH on cAMP (**F**) or pERK (**G**) pathways on WT and variants G89R, R151C and D294H. Data represent mean ± SEM of *n* = 2. Efficacy (**H**) for cAMP and pERK responses were analysed using log(agonist) vs. response - variable slope (four parameters model).

We then determined the functional consequences of variants on eight additional melanocortin agonists, including approved and investigational drugs, which present agonistic activity at wild type MC_1_ at one or both pathways (Fig. 3A–C). The simultaneous analysis of the effect of these ligands on all variants (Fig. 3D, E, S6,7) reveals receptor areas which are crucial for the activation of cAMP: all drugs, independently on their nature (peptide, small molecule), affinities or receptor subtype preference, required the positions A81, S83, D84 and G89 (‘block 1’, Fig. 3D) within TM2 domain to be intact in order to activate the receptor. Another sensitive region is found at the IC2 domain and surrounding area (‘block 2’: R142, A149, R151, I155, R160, Fig. 3D). Other critical positions include R213, L252, N279, D294 and R306. All except for one RHC variant (T95M) sit in these critical positions. LoF at block 1 and D294H was also observed for pERK signalling (Fig. 3E). However, this was not the case for block 2, in which the clear LoF at cAMP for all drugs was no longer occurring for pERK. This suggests that the molecular determinants required to engage with each pathway may be different, which is congruent with the existence of ligand bias on this receptor (Fig. S8). Indeed, the correlation between cAMP and pERK differs substantially for most compounds (Fig. S9A). Importantly, the activity of ⍺MSH partially reflects the behaviour of the other compounds, as the correlation was high, but only for cAMP and not for pERK (Fig. S9B, C).

Next, we performed a validation of the screening above by generating full concentration-response curves on three selected variants, G89R, R151C and D294H, as exemplars of variants of ‘block 1’, ‘block 2’ and an additional variant in domain TM7, respectively. We confirmed that G89R and D294H cause drastic LoF at both pathways and that R151C presents with prominent LoF at cAMP but full agonistic activity at pERK (Fig. 3F–H).

### In vitro anti-inflammatory responses reveal the existence of responders and non-responders to melanocortin agonists

**Fig. 4:**
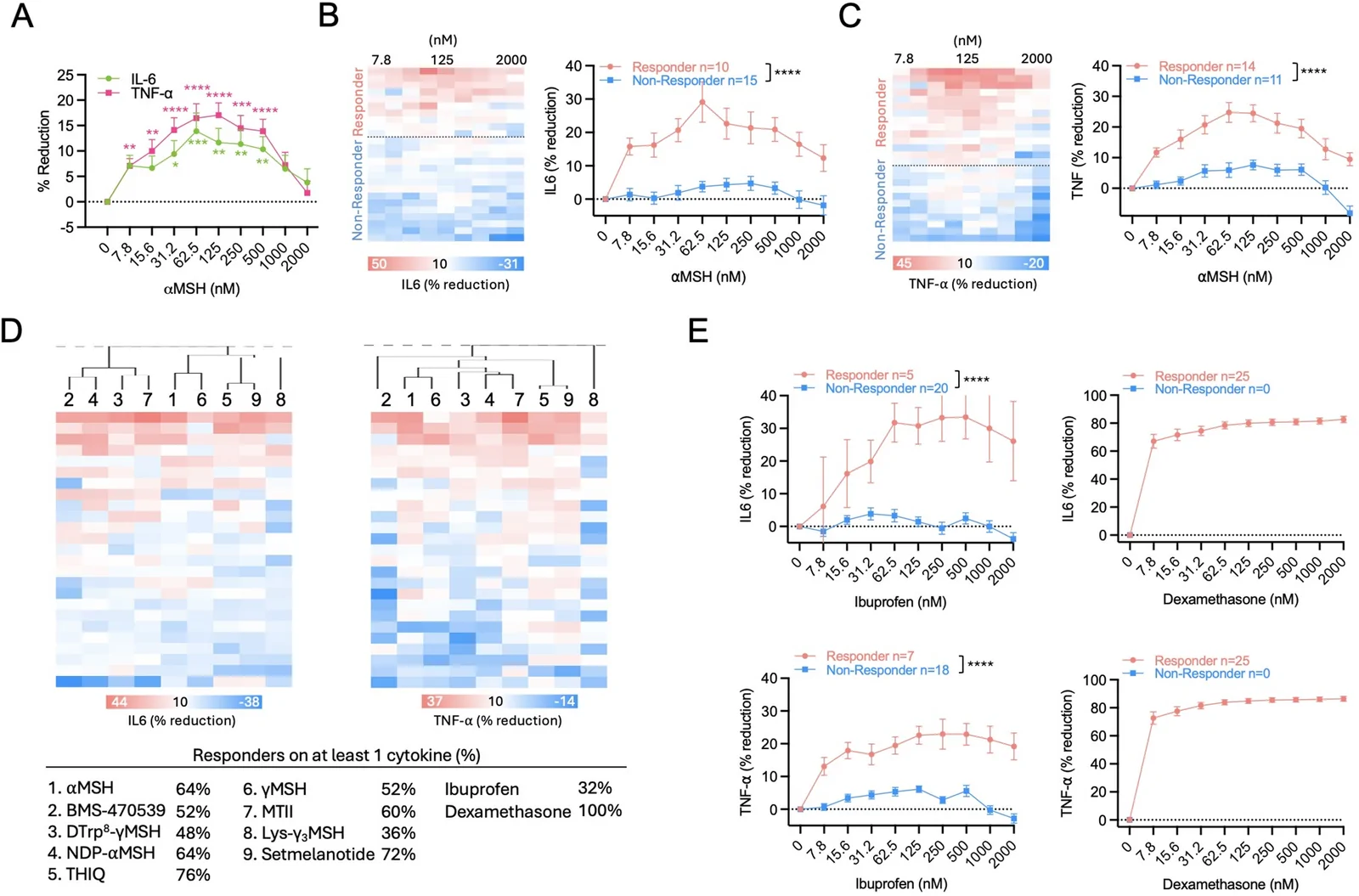
Anti-inflammatory efficacy of melanocortin compounds on the whole blood assay. Diluted whole blood was treated for 30 min with melanocortin drugs (7.8–2000 nM) before being stimulated with 1 µg/ml LPS. Samples were collected 3 h later and cytokine release analysed by ELISA. Vehicle (PBS), dexamethasone and ibuprofen were used as controls. Data for all donors and compounds is normalised as % of reduction in cytokine content with respect to vehicle control. (**A**) Reduction in IL6 and TNF-⍺ release by ⍺MSH. (**B**) Reduction in IL6 release by ⍺MSH, for each of the 25 donors, classified as responders (red) and non-responders (blue). (**C**) Reduction in TNF-⍺ release by ⍺MSH, for each of the 25 donors, classified as responders (red) and non-responders (blue). (**D**) Reduction on IL6 and TNF-⍺ release by each of the nine melanocortin compounds tested. Heat map is displayed according to hierarchical clustering (one minus Pearson correlation) of the compounds. (**E**) Reduction in IL6 and TNF-⍺ release by ibuprofen and dexamethasone, for each of the 25 donors, classified as responders (red) and non-responders (blue). Data represent mean ± SEM of *n* = 25 donors, each tested in duplicates. Data represent mean ± SEM and was analysed by paired one-way ANOVA with Dunnett’s multiple comparisons test using the original non-transformed values (i.e. pg/ml) in A, and by paired two-way ANOVA (responders vs. non-responders) in B, C,E; *p < 0.05, **p < 0.01, ***p < 0.001, ****p < 0.0001.

We next evaluated the anti-inflammatory actions of melanocortin compounds. Whole blood was obtained from 25 healthy volunteers and stimulated with lipopolysaccharide (LPS); IL6 and TNF-⍺ were assessed by ELISA after 3 h. A clear bell-shaped response was observed for ⍺MSH (Fig. 4A) on both mediators. Maximal efficacy occurred at 62.5–125 nM, reaching 13.9% (IL6) and 17% (TNF-⍺) reduction, on average. However, the analysis at individual level reveals the existence of responders and non-responders. By setting a cut-off criteria of ≥ 10% reduction (calculated as the average from 15.6 to 1000 nM, to exclude the bell-shape effect), the efficacy on the responders group increased to 29% (IL6) and 24.8% (TNF-⍺) (Fig. 4B, C). Eight additional melanocortin compounds were tested, observing again a bell-shape pattern, interestingly also for ibuprofen (Fig. 4D, S10A) and in most cases the responses to IL6 and TNF-⍺ were significantly correlated (Fig. S10B). The most effective of all melanocortin compound was ⍺MSH. We identified responders and non-responders for all compounds (Fig. 4D, S11), and the response appear consistent across donors. For example, donors 1, 8 or 13 responded to most ligands, while donors 5, 11 or 12 hardly showed any response to any compound. The response to immunosuppressive drug dexamethasone was sharp, with 100% penetrance and an almost identical degree of inhibition (70–80%) with as little as 7.8nM (Fig. 4E).

### Loss-of-function variants at MC1R reduce the anti-inflammatory activity of melanocortin compounds in vitro

**Fig. 5:**
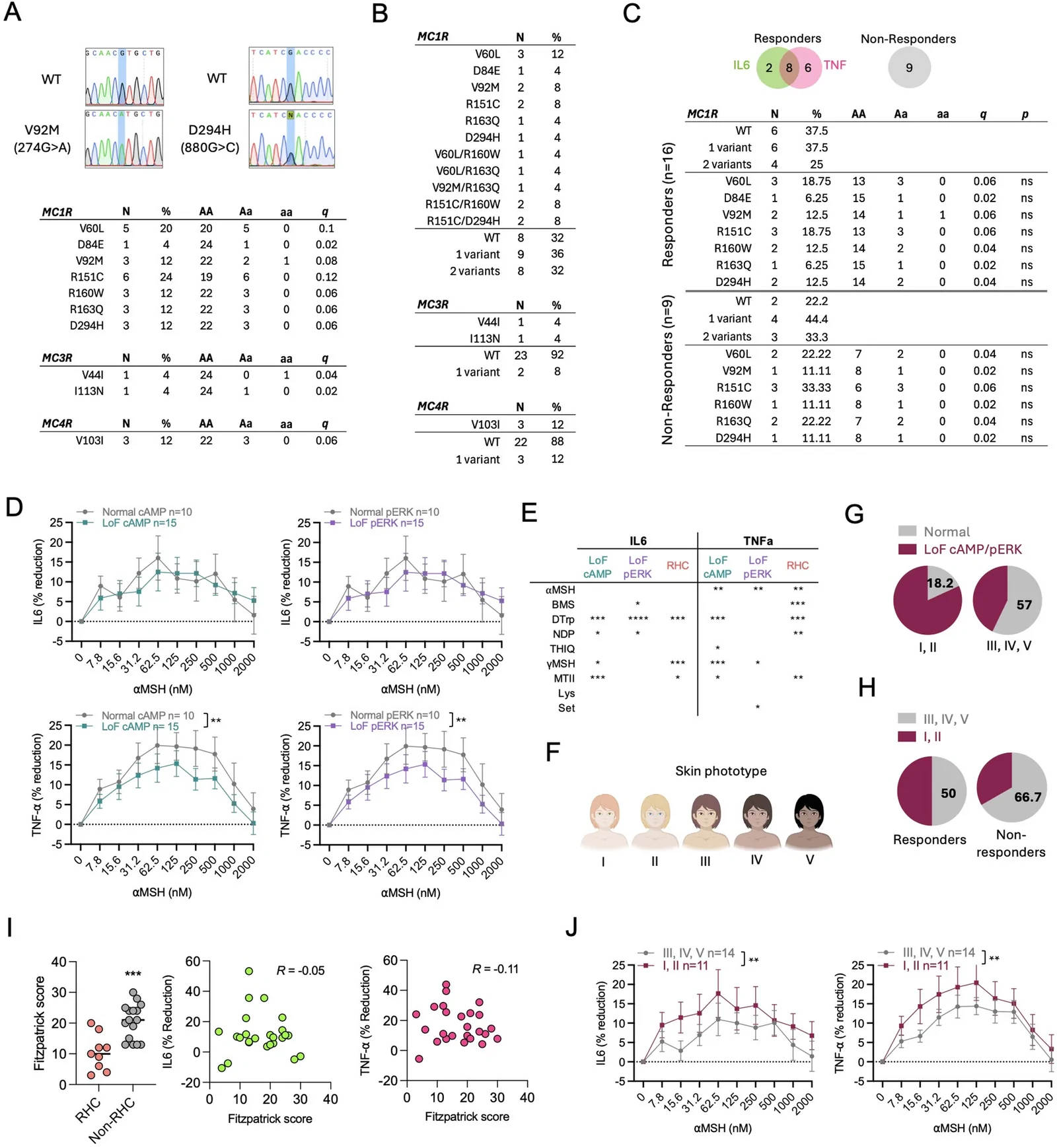
Effect of *MC1R* variants on cytokine responses to melanocortin compounds in the whole blood assay. (**A**) *MC1R*,* MC3R* and *MC4R* genotyping of the 25 healthy volunteers. Examples of chromatograms showing the detection of V92M and D294H variants on *MC1R*. N: nucleotide, %: proportion of carriers of each variant, AA: number of donors homozygous for mayor allele (i.e. WT), Aa: number of donors heterozygous, aa: number of donors homozygous for the minor allele (i.e. variant), *q*: minor allele frequency. (**B**) Haplotypes identified; number (N) and percentage (%) of donors with a variant or combination of variants. (**C**) Analysis of *MC1R* variants on responders or non-responders to ⍺MSH at least at one cytokine, *p*: Fisher’s exact test of responders vs. non-responders. ‘Responder’ is defined as reaching ≥ 10% reduction on cytokine levels considering the average from 15.6–1000 nM. (**D**) IL6 and TNF-⍺ responses to ⍺MSH based on LoF variants at cAMP and pERK. (**E**) IL6 and TNF-⍺ responses to all melanocortin compounds based on LoF variants at cAMP, pERK or presence of an RHC variant. Full graphs and 95% CI reported in Figs. S12–14. (**F**) Schematic representing the skin phototypes resulting from the Fitzpatrick questionnaire. (**G**) Association between skin phototypes and the presence of LoF variants at *MC1R*. (**H**) Association between skin phototypes and response to ⍺MSH (‘responder’ defined as reaching ≥ 10% reduction on cytokine levels considering the average from 15.6-1000nM). Numbers in the pie charts indicate the percentage of individuals. (**I**) Association between Fitzpatrick score with the presence of a red hair variant (RHC), reduction in IL6 or in TNF-⍺ by ⍺MSH at 125 nM. (**J**) Concentration-response of ⍺MSH for IL6 and for TNF-⍺ based on skin phototypes III, IV or V (grey) of skin phototypes I or II (plum). Full graphs and 95% CI reported in Fig. S16. Data represent mean ± SEM and was analysed by two-way ANOVA (**D**,**E**,**J**), Fisher exact test (**G**), or by t-test and Pearson correlation test (**I**); *p < 0.05, **p < 0.01, ***p < 0.001, ****p < 0.0001.

Next, we addressed the influence of *MC1R* variants on the anti-inflammatory activity of melanocortin agonists. We also analysed *MC3R* and *MC4R* genes, given their importance in driving anti-inflammatory actions. We detected seven variants at *MC1R*, with R151C being the most frequent (24%, Fig. 5A). A third (32%) of donors carried the WT form of *MC1R*, while 68% carried at least one variant (Fig. 5B). We then stratified participants into responders to ⍺MSH on at least one cytokine (*n* = 16) and non-responders at both cytokines (*n* = 9). Although carrying a variant at *MC1R* is common (68%), the frequency of each individual variant is generally too low to establish associations (Fig. 5C). Only two and three donors presented with a variant in *MC3R* and *MC4R*, respectively, discarding any contribution from these genes.

However, when grouping variants together based on similar signalling responses, associations were identified. We analysed responders and non-responders to all nine melanocortin compounds, based on whether they carried (i) a LoF variant at cAMP, (ii) a LoF variant at pERK or (iii) an RHC variant (Fig. 5D, for ⍺MSH, full curves in Fig. S12-14). We found statistically significant reduction in the anti-inflammatory activity in LoF carriers for all compounds tested except for Lys-γ_3_MSH (Fig. 5E), at least in one instance (IL6 or TNF-⍺, for cAMP, pERK or RHC), strongly suggesting that impaired *MC1R* signalling results in diminished anti-inflammatory activity. The most pronounced effects were observed for DTrp8-γMSH, with an IL6 response 2.3 times higher in WT at 250 nM, and the lowest, for setmelanotide, which was only minimally affected by the presence of variants.

### Skin phototype does not predict anti-inflammatory activity of melanocortin compounds

To conclude this work, we aimed to address if a simple assessment like the Fitzpatrick questionnaire, used to evaluate skin phototype (Fig. 5F, Fig. S15), could predict anti-inflammatory response of melanocortin agonists. As predicted, darker skin types (III-V) were more likely carriers of the WT or a variant not associated with LoF (57%), while lighter skin types (I, II), mostly carried a LoF variant at *MC1R* (Fig. 5G). Unexpectedly, when analysing the response to ⍺MSH, darker skin types were more frequent in non-responders (Fig. 5H).

Congruently, lower Fitzpatrick scores were associated with carrying a RHC variant, but not with the inhibition of neither IL6 nor TNF-⍺ (Fig. 5I). We then stratified donors based on light (I, II), or darker (III-V) skin phototypes for ⍺MSH. While significant differences were found, results appear inconsistent, as higher reduction on IL6 and TNF-⍺ release was obtained for donors with lighter skin types (Fig. 5J). A similar trend was observed for several other melanocortin compounds (Fig. S16), altogether suggesting that Fitzpatrick scores and derived phototype, cannot be used as predictors of responsiveness, as factors other than *MC1R* variant contribute this score (e.g. other genes, UV exposure behaviour, etc.).

## Discussion

*One-size-fits-all* and *trial-and-error* approaches currently dominate the therapeutic management of most diseases. This is, not only ineffective but also detrimental in many cases, leading to poor clinical outcomes, side effects, delayed interventions and drained healthcare systems resources. Therefore, being able to predict in advance which patients will respond to a given therapy (i.e. the effective application of ‘precision medicine’) could have a profound impact at many levels. This is what we aimed to address in the present work, focusing on a new class of anti-inflammatory drugs targeting the melanocortin 1 receptor (MC_1_), a GPCR encoded by an unusually polymorphic gene, *MC1R*.

The genetic diversity at *MC1R* is vast: 900 variants in the coding region were identified, spanning almost the totality of amino acids (91%) comprising the receptor. Regardless of which intriguing mechanisms during the evolution of the *Homo sapiens* (migrations, admixture events, founder effects, genetic drift, culture, etc.) resulted in this diversity, the fact that MC_1_ is a therapeutic target, makes the elucidation of the functional consequences of variants highly relevant. The analysis of cAMP and pERK pathways in thirty selected variants reveals a ‘continuum’ of effects, from GoF, no effect, mild and moderate LoF and even complete abrogation of receptor activity at both pathways. This gradual, non-discrete effect may explain why common predictor tools (Polyphen, SIFT, and others) generally failed to accurately determine pathogenicity at this receptor, when interpreted as binary outcomes (i.e. either ‘pathogenic’ or ‘benign’), while showing relatively good correlation with signalling responses when we use the numerical scores instead. The term ‘pathogenicity’ of a genetic variant is also vague, particularly when applied to genes with complex functionalities like GPCRs (e.g. signalling networks, kinetics, biased signalling, intrinsic activity), or when LoF is indeed a gradual phenomenon, rather than a yes/no (i.e. binary outcome). How do we define pathogenicity at *MC1R*? LoF at cAMP? At pERK? At both pathways simultaneously? Based on red hair association? Our data establish the need for functional experiments to determine the impact of genetic variants on receptor activity, since at present, reliance on in silico predictor tools is not reliable to implement pharmacogenetics.

Another important question is whether ⍺MSH responses could predict the response by other agonists. Strong similarity on how variants affect each of the nine compounds tested was observed, mostly on cAMP activity, revealing well-defined areas at TM2 and IC2 domains, and additional positions like D294H. The intrinsic activity followed a pattern similar to that induced by the compounds, suggesting that the diminished function caused by the variants may not be related to aspects like ligand binding. Elucidation of the crystal structure of MC_1_ has provided invaluable insights into the mechanisms involved in receptor activation. Ma et al.^16^ reported that variants can differentially affect the molecular recognition of different peptides: incorporation of alanine at position D117 severely impairs receptor activation by ⍺MSH while hardly affects activation by NDP-⍺MSH. Strikingly, introducing an alanine in M128 increases the potency of the antagonist SHU9119, while a leucine at the same position turns this compound into an agonist. Although the D117 and M128 variants are not naturally found in the population, these data highlight the complex interplay between both receptor requirements and ligand requirements in determining the final outcome, emphasizing once more the difficulty in predicting the efficacy of melanocortin drugs in carriers of variants. It is important to consider that cells in a physiological environment may display differences in signalling and receptor expression compared with heterologous systems, as used in this study, and further studies may be required to confirm the observed outcomes.

Our study also brings up the plausible dissociation between cAMP and pERK pathways. GoF was rare at cAMP but frequent at pERK. The correlation between both pathways was also generally low. Furthermore, the strong LoF at cAMP observed with variants at ‘block 2’ was completely absent at pERK. Then, while an intact ‘block 1’ seems to be required for the engagement of both pathways, variations in ‘block 2’ only impaired cAMP activation. Garcia-Borron and colleagues have extensively studied regulatory mechanisms underpinning MC_1_ receptor activation. They reported that pERK was dependent on cAMP in mouse but not in human melanoma cells^17^ and that competitive interactions between different β-arrestin proteins exert differential control over cAMP and pERK^18^. Our data illustrates that not all RHC variants alter the receptor in the same way, even if all result in red hair phenotype. Eumelanin production is fully dependent on cAMP^19,20^, hence the association of cAMP LoF variants R151C or D294H with red hair. However, some RHC variants retain their ability to stimulate pERK (e.g. R142H, R151C, R160W - ‘block 2’) while others don’t (S83P, D84E -block 1’).

The translational implications of these findings are that functional outcomes derived from pERK might be retained for example in R151C but lost in D294H carriers. However, which functional outcomes result form MC_1_-derived pERK are still to be elucidated. Interestingly, it was reported that nephrotic syndrome patients carrying the variant R151C responded to ACTH therapy^21^ leading the authors to conclude that MC_1_ was dispensable for the actions of ACTH. Our own data may provide another explanation: the retained activity at pERK by R151C may have contributed to the therapeutic effect of this melanocortin agonist.

We then addressed the impact of *MC1R* variants on the anti-inflammatory potential of melanocortin compounds, using the whole-blood assay, as a simple mid-throughput screening model. Overall responses were similar to other reported MC compounds^22^. Responders and non-responders were clearly identified. When taking into account the presence of LoF variants, a significant association was found for most compounds. Our data implicate cAMP engagement in the anti-inflammatory actions of MC molecules although we cannot discard the contribution of pERK. Our cohort did not include carriers of variants with LoF mostly at pERK (e.g. V97I, V122M), thus we could not explore the contribution of this pathway further. The fact that all variants were found in heterozygous state and that other receptors likely contribute to the anti-inflammatory actions (e.g. MC_3_, MC_4_) may explain why anti-inflammatory activity, albeit diminished, was still observed in *MC1R* LoF variants carriers. Congruently, the highest proportion of responders, 76% and 72%, was found for THIQ and setmelanotide respectively, both compounds with a strong preference for MC_4_^23,24^.

In terms of the feasibility of using ‘red hair’ or skin phototype as a predictor, we propose that this is not a sensible way forward. Many other genes are also associated with pigmentation and red hair, like *ASIP*, *HERC2*, *TSPAN10* and notably *POMC*^12,25,26^, the gene encoding for the precursor of melanocortin ligands. Only 5 out of 9 RHC carriers in our cohort had red hair, and they carried two independent heterozygous RHC. This implies that carrying an RHC variant does not directly translate into having red hair. Of note, our data also suggest that several variants currently not described as RHC might indeed be so. Based on their signalling profile, we may speculate that A81P, G89R, A149T or L252H may also result in red hair.

In conclusion, the data presented here strongly point to the need to incorporate pharmacogenetics into melanocortin drug development, and likely any programs in which the target is a GPCR. Currently, CXCR4 is the only GPCR included in the FDA table of pharmacogenomics biomarkers. The decision of which patient may or may not receive a treatment requires having clear-cut evidence-based data on gene-drug interactions. As we argue here, such clear-cut evidence cannot be obtained from in silico predictors, as the impact of variants depend, not only on the specific variant that the patient carries, but on the specific drug the patient may receive. We hope this work awakens the need among drug developers to incorporate studies on the pharmacological impact of *MC1R* variants to produce more effective and safer drugs.

## Materials and methods

### Public data repositories and analysis tools

The study of human *MC1R* genetic diversity was performed using Ensembl browser. Genetic variation data for *MC1R* gene (ID: ENSG00000258839) was downloaded in April 2020 and used to obtain the variants and pathogenicity scores at the start of this study. A total of 900 variants in the coding region were obtained. Then, synonymous, COSMIC variants and other variants for which pathogenicity data is not available (e.g. stop codons) were excluded, yielding 428 variants that were used for further analysis. Only predictions unequivocally identified as pathogenic/deleterious by the in silico predictor algorithms obtained from Ensembl and AlphaMissense were considered as such, i.e. with scores as follows: SIFT < 0.05, PolyPhen > 0.908, REVEL > 0.5, MelalR > 0.5, Mutation Assessor > 0.5, AlphaMissense > 0.56. GPCRdb (March 2025 release^27^) was used to obtain a snake plot for the MC_1_ protein structure and visualise the selected variants. Genome Aggregation Database (gnomAD, v2.1.1^28^) was used to obtain allele frequency information. AlphaMissense (version October 2023) was used to obtain pathogenicity predictions for all amino acid positions of the MC_1_ sequence, and for all possible amino acid changes at each position^15^.

### Determination of predictive values

The measures used to evaluate the predictors tools were defined as follows^29^: positive predictive values (PPV) - proportion of variants testing pathogenic that are truly pathogenic (true positive/(true positive + false positive); negative predictive value (NPP) - proportion of variants testing benign that are truly benign (true negative/(true negative + false negative); sensitivity - ability to correctly classify a variant as pathogenic (true positive/(true positive + false negative); specificity - ability to correctly classify a variant as benign (true negative /(true negative + false positive); accuracy - proportion of cases (both benign and pathogenic) correctly identified (correct predictions / total predictions).

### Compounds

The following melanocortin ligands were used for pharmacological assays: αMSH (Tocris Bioscience, Cat#2584); γ_1_MSH (MedChemExpress, Cat#HY-P1531); [D-Trp8]-γMSH (Cayman Chemical, Cat#14037); Lys-γ_3_MSH (Tocris Bioscience, Cat#3611); THIQ (Tocris Bioscience Cat#3032); melanotan II (Tocris Bioscience, Cat#: 2566,); [Nle4, D-Phe7]-αMSH (Tocris Bioscience, Cat#3013); BMS-470,539 hydrochloride (Cayman Chemical, Cat#CAY22231); and setmelanotide (Selleckchem, Cat#E1002). Other compounds include: 3-isobutyl-1-methylxanthine (IBMX, Sigma-Aldrich, St. Louis, USA, Cat#I5879), forskolin (Tocris Bioscience, Cat#1099/10), lipopolysaccharide from *E. coli* O111:B4 (Sigma-Aldrich, Cat#L2630), ibuprofen sodium salt (Sigma-Aldrich, Cat#I1892) and dexamethasone (Sigma-Aldrich, Cat#D4902).

### Preparation of the MC1R variants plasmid collection

Human MC1R TrueORF^®^ clones (wild-type and selected variants) in the pCMV6-Entry vector with C-terminal Myc-DDK tags were obtained from OriGene through the custom clone modification service (OriGene Technologies, Inc, Cat# RC203218). Lyophilized plasmids (10 µg) were reconstituted in 100 µl sterile water and transformed into *E. coli* JM109 competent cells (Promega Corporation, Cat#L2001) by heat shock following the manufacturer’s instructions. Selected colonies were cultured in 6 ml LB medium supplemented with 25 µg/ml kanamycin, and plasmid DNA was isolated using the Zyppy Plasmid Miniprep Kit (Zymo Research Corporation, Cat#D4019). DNA sequence was confirmed by Sanger sequencing using OriGene primers VP1.5 (5′-GGACTTTCCAAAATGTCG-3′) and XL39 (5′-ATTAGGACAAGGCTGGTGGG-3′) at Eurofins Genomics (Ebersberg, Germany) sequencing service. Verified clones were re-grown in 70 ml LB medium supplemented with 25 µg/ml kanamycin and purified using Qiagen HiSpeed Plasmid Midi Kit (Qiagen, Cat#12145). Stable glycerol stocks of all clones were prepared and stored at − 80 °C.

### Cell culture and transient transfection

HEK293 cells (ATCC^®^ CRL-1573™, American Type Culture Collection, Manassas, USA) were maintained in DMEM, high glucose, no glutamine, no phenol red (Thermo Fisher Scientific, Cat#31053-028) supplemented with 10% heat-inactivated foetal bovine serum (Thermo Fisher Scientific, Cat#A5256801), 100 U/ml penicillin + 100 µg/ml streptomycin (Sigma-Aldrich, Cat#P4333), and 2 mM L-glutamine (Sigma-Aldrich, Cat#G7513), at 37 °C/5% CO₂. Cells were sub-cultured every 7 days at a 1/30 split ratio. For transient transfection, cells were seeded at 15,000 cells/well in 96-well plates (Corning Inc., Cat#3599) and transfected 24 h later with 100 ng plasmid DNA of the corresponding *MC1R* variant or empty pCMV6 vector and 0.2 µl Lipofectamine^®^ 2000 (Thermo Fisher Scientific, Cat#11668-019) per well in Opti-MEM™ I Reduced Serum Medium (Thermo Fisher Scientific, Cat#31985-062). Assays (cAMP and pERK signalling) were performed 24 h post-transfection.

NIH 3T3 cells (ATCC^®^ CRL-1658™, ATCC, Manassas, USA) were maintained in DMEM (Thermo Fisher Scientific, Cat#31053-028) supplemented with 10% heat-inactivated foetal bovine serum (Thermo Fisher Scientific, Cat#A5256801), 100 U/ml penicillin + 100 µg/ml streptomycin (Sigma-Aldrich, Cat#P4333), and 2 mM L-glutamine (Sigma-Aldrich, Cat#G7513) at 37 °C/5% CO₂. Cells were sub-cultured every 7 days at a 1/20 split ratio. For transient transfection, cells were seeded at 50,000 cells/well in 48-well plates (Corning Inc., Cat#3548) and transfected 24 h later with 400 ng plasmid DNA and 0.75 µL Lipofectamine^®^ 2000 (Thermo Fisher Scientific, Cat#11668-019) per well in Opti-MEM™ I Reduced Serum Medium (Thermo Fisher Scientific, Cat#31985-062). Cells were subjected to immunofluorescence 24 h post-transfection.

### Screening design

The screening was performed in 96-well plate format. To account for batch effects and potential differences in cell growth and expression levels across experiments, all variants for each of the compounds were tested always on the same plate, which included empty vector transfected cells (to account for basal activity on each batch) as well as wild type *MC1R* transfected cells treated with vehicle. All data is always expressed as % of wild type referred to its own internal control included in each plate. Transfection efficiency was determined by immunofluorescence against the Myc-Tag included in each clone. For wild type *MC1R*, efficiency was 31.1% and the average for *MC1R* variants 26.3 ± 6.2 (mean ± SD).

### cAMP accumulation assay

Twenty-four hours after transfection with the corresponding *MC1R* variant, the culture medium was aspirated and replaced with 50 µl of serum-free medium. Cells were serum-starved for 2 h at 37 °C/5% CO₂ to stabilize basal cAMP levels. Then, the culture medium was removed and 150 µl/well of test compounds prepared in serum-free medium containing 1 mM IBMX (3-isobutyl-1-methylxanthine) were added. For *MC1R* variants screening, melanocortin compounds were tested at 10 µM, while for concentration–response curves, melanocortin agonists were tested using 1/5 serial dilutions starting at 10 µM. Vehicle (PBS) and 3 µM forskolin were included as negative and positive controls, respectively. After 15 min stimulation, reactions were terminated by placing plates on ice, adding 7.5 µl/well of 2 N HCl, and scraping cells using a multichannel pipette. Plates were frozen at − 80 °C until processing by ELISA. cAMP levels were quantified using the Cyclic AMP Select ELISA Kit (Cayman Chemical, Cat#: CAY501040-480) according to the manufacturer’s instructions. Cell lysates were thawed, briefly centrifuged, diluted 1:1 in EIA buffer and 50 µL used for the assay. *MC1R* variant screening assays were performed in triplicate, while full concentration–response curves for selected variants were run in duplicates.

### ERK1/2 phosphorylation assay

Twenty-four hours after transfection with the corresponding *MC1R* variant, the culture medium was aspirated and replaced with 50 µl of serum-free medium. Cells were serum-starved for 2 h at 37 °C/5% CO₂ to stabilize basal phospho-ERK1/2 levels. Test compounds were prepared at 2X concentration in serum-free medium and 50 µl added directly to wells (containing 50 µl of medium). For *MC1R* variants screening, melanocortin compounds were tested at 10 µM. Concentration–response curves were generated using 1:5 serial dilutions starting at 10 µM. Cells were stimulated for 5 min, with timing synchronized across all wells. Following stimulation, the medium was removed, and the plates were placed on ice. Cells were lysed immediately with 90 µl of ice-cold Extraction Buffer provided in the Phospho-ERK1/2 (Thr202/Tyr204) SimpleStep ELISA Kit (Abcam, Cat#ab176640) and detached by scraping with a multichannel pipette. Lysates were briefly centrifuged, and pERK levels were quantified according to the manufacturer’s protocol. *MC1R* variant screening assays were performed in triplicate, while full concentration–response curves for selected variants were run in duplicate at each concentration.

### Immunofluorescence

NIH 3T3 cells were seeded on sterile glass coverslips in 48-well plates and transfected as described above. At 24 h post-transfection, culture medium was gently removed, and cells were fixed in 4% paraformaldehyde in PBS (Santa Cruz, Cat#sc-281692) for 20 min at room temperature. Fixed cells were washed three times for 10 min in PBS on a low-speed shaker, permeabilized for 10 min with 0.3% Triton X-100 (Merck, Cat#X100) in PBS and blocked for 60 min in blocking buffer (5% normal goat serum (Abcam, Cat#ab7481), 0.3% Triton X-100 in PBS). Cells were incubated overnight at 4 °C in the dark with anti-Myc Tag (9B11) Alexa Fluor^®^ 594 conjugate (clone 9B11, Cell Signaling Technology, Cat#9483) diluted 1:100 in assay buffer (5% normal goat serum, 0.3% Triton X-100 in PBS). Alexa Fluor^®^ 594 Mouse IgG2a, κ isotype control (clone MOPC-173, BioLegend, Cat#400280) was used for control staining. The following day, cells were washed three times for 10 min in PBS, and coverslips were mounted on microscopy slides using ProLong™ Diamond Antifade Mountant with DAPI (Invitrogen, Cat#P36971) and slides were allowed to cure overnight at room temperature in the dark. Imaging was performed using a Zeiss LSM 880 confocal microscope with Airyscan, using the 561 nm laser line for Alexa Fluor^®^ 594 and the DAPI channel for nuclei. Screening images were acquired at 63X with Zoom 0.8 (~ 50X).

### Recruitment of healthy volunteer blood donors and ethical approval

The study was approved by the Queen Mary Ethics of Research Committee (Ref QMU24.0360, Queen Mary University of London). All methods were performed in accordance with relevant guidelines and regulations, including Declaration of Helsinki and Good Clinical Practice. Healthy volunteers aged ≥ 18 years, non-pregnant, medication-free for at least 7 days prior to blood donation, and who had not donated blood within the preceding 7 days were invited to participate. Written informed consent was obtained from all participants prior to sample collection. Demographic data, including age, sex, ethnic background, and responses to a self-administered Fitzpatrick skin type questionnaire, were collected.

### Whole blood assay

Blood samples from healthy volunteers were collected into 3.2% sodium citrate in PBS (Sigma-Aldrich, Cat#S4641) at a 1:10 ratio, gently mixed, and diluted 1:5 in pre-warmed RPMI (Thermo Fisher Scientific, Cat#32404014). 15 µl of test compounds or vehicle (PBS) were prepared in a separate 96-well U-bottom plates at 10X final concentration in RPMI medium. Then, 150 ul of diluted blood was added to each well containing test compounds, and plates were incubated for 30 min at 37 °C/5% CO₂. Lipopolysaccharide (LPS) solution was added at a final concentration of 1 µg/ml. Unstimulated control wells received RPMI medium. After 3 h at 37 °C/5% CO₂, plates were centrifuged at 2,000 rpm for 5 min at room temperature. Supernatants were collected and stored at − 80 °C until analysis by ELISA. All assays were performed in duplicates, and unstimulated (i.e. no LPS) and vehicle controls were included in every plate. The melanocortin ligand panel tested included αMSH, BMS-470,539, [D-Trp8]-γMSH, NDP-αMSH, melanotan II, THIQ, γMSH, Lys-γ_3_MSH, and setmelanotide. Anti-inflammatory drugs dexamethasone and ibuprofen were included as positive controls. Compounds were tested using 1/2 serial dilutions in RPMI starting at 2 µM.

### Cytokine determination by ELISA

Supernatants obtained from the whole blood assay were analysed for pro-inflammatory cytokines using Human IL6 and TNF-α Uncoated ELISA kits (Thermo Fisher Scientific, Cat#88-7066 and 88-7346) according to the manufacturer’s instructions. Samples were diluted in ELISA/ELISPOT Diluent (1:20 for IL6 and 1:10 for TNF-α; dexamethasone-treated samples at 1:10 for IL6 and 1:5 for TNF-α). Plates were developed with TMB substrate, stopped with 1 M H₂SO₄, and absorbance read at 450 nm. A standard curve was included in each assay for the absolute quantification using linear standard curve interpolation.

### Genomic DNA extraction

Genomic (g)DNA was extracted from 100 µl of whole blood using Monarch^®^ Spin gDNA Extraction Kit (New England Biolabs, Cat#T3010S) according to the manufacturer’s protocol. The gDNA was eluted with 75 µl of RNase/DNase-free water. Quantity (ng/µl) and purity (260/280 and 260/230 ratios) was measured using a NanoDrop™ 2000 (Thermo Fisher Scientific).

### MC1R gene genotyping and analysis

The coding region of human *MC1R*, *MC3R* and *MC4R* were amplified using in-house designed primers, obtained from Merk oligo synthesis service. *MC1R* was sequenced in two overlapping fragments, using the in-house designed primers forward: 5’-GCCCAGATGGAAGGAGGC-3’ and reverse: 5’-CATGAGCACCAGCATAGCCA-3’ (fragment 1) and forward: 5’-CTGCTTCCTGGGCGCCAT-3’ and reverse: 5’-CAGAGATCATTTAGTCCATCCTC 3’ (fragment 2). PCR cycling conditions were 95 °C for 1 min, followed by 34 cycles of 95 °C/15 s, 65 °C/20 s, and 72 °C/30 s, with a final extension at 72 °C for 5 min. For *MC3R*, primers were forward 5′-ATCCAAAAGACGTATCTGGAGGG-3′ and reverse 5′-TTTCTAAACACCTCACGTGGATG-3′, with cycling at 95 °C for 1 min, followed by 34 cycles of 95 °C/15 s, 61 °C/20 s, and 72 °C/50 s, and a final extension at 72 °C for 5 min. *For MC4R*, primers were forward 5′-ACTTGGAGGAAATAACTGAGACG − 3′ and reverse 5′-GGAAGAGAAAGCTGTTGCAGAAG-3′, with cycling at 95 °C for 1 min, followed by 34 cycles of 95 °C/15 s, 62 °C/20 s, and 72 °C/50 s, and a final extension at 72 °C for 5 min. 50 ng of gDNA was amplified using the TaKaRa Taq PCR kit (TaKaRa, Cat#639209) with 0.6 mM NEB dNTPs (New England Biolabs, Cat#N0447S). PCR products were purified and subjected to Sanger sequencing at Eurofins Genomics service (Ebersberg, Germany). Sanger sequencing was performed using ABI 3730xl DNA Analyzer and basecaller KB v1.4.1.8. Data obtained as *ab1 files were analysed using MacVector with Assembler (v18.7.8) against the *Homo sapiens* reference sequences NC_000016.10 (*MC1R*), NM_019888 (*MC3R*), and NM_005912.3 (*MC4R*).

### Statistical analysis

Specific statistical test, number of replicates and p values are indicated in each figure legend.

Briefly, the screening experiments of variants were performed in triplicates and analysed by one-way ANOVA with Dunnett’s multiple comparisons test and by Pearson correlation test. Concentration–response curves for cAMP and pERK assays (conducted in duplicates) were analysed using an operational model - partial agonist, X is log(concentration). Potency (EC_50_) values were obtained from normalised data using log(agonist) vs. response - variable slope (four-parameter model). The whole blood assay was conducted in 25 donors (*n* = 25) each one tested in duplicates. Data was analysed by paired two-way ANOVA (responders vs. non-responders) and by paired one-way ANOVA with Dunnett’s multiple comparisons test of each concentration vs. vehicle group, using the original non-transformed values (i.e. pg/ml). Hierarchical clustering analyses were performed by one minus Pearson correlation, with Morpheus matrix visualization and analysis software. Association of variants or skin phototype with cytokines responses was analysed by Fisher’s exact test and two-way ANOVA. Statistical analysis was performed with GraphPad Prism 10 for macOS, v 10.6.0 (796). Significance thresholds: *p < 0.05, **p < 0.01, ***p < 0.001, ****p < 0.0001.

## Supplementary Information

Below is the link to the electronic supplementary material.

Supplementary Material 1

## Data Availability

The sequencing datasets generated and analysed during the current study are available in the Figshare repository, [https://doi.org/10.6084/m9.figshare.31586662]. All other data supporting the findings of this study are available within the article, its Supplementary Data files, and from the corresponding author upon reasonable request.
