# Supplementary material for: The anti-inflammatory efficacy of melanocortin drugs is influenced by genetic variation at MC1R
Source: Sci Rep. 2026 Jun 11;16:26756. doi: 10.1038/s41598-026-57399-0 (PMC13507197; doi:10.1038/s41598-026-57399-0)
Supplement: Supplementary file 1 [file 41598_2026_57399_MOESM1_ESM.pdf]

Supplementary Data

The anti-inflammatory efficacy of melanocortin drugs is influenced by genetic variation at *MC1R*

Natalya Khodeneva, Camilla SA Davan-Wetton, Thomas EN Jonassen, Mauro Perretti,

Trinidad Montero-Melendez

Fig. S1

A

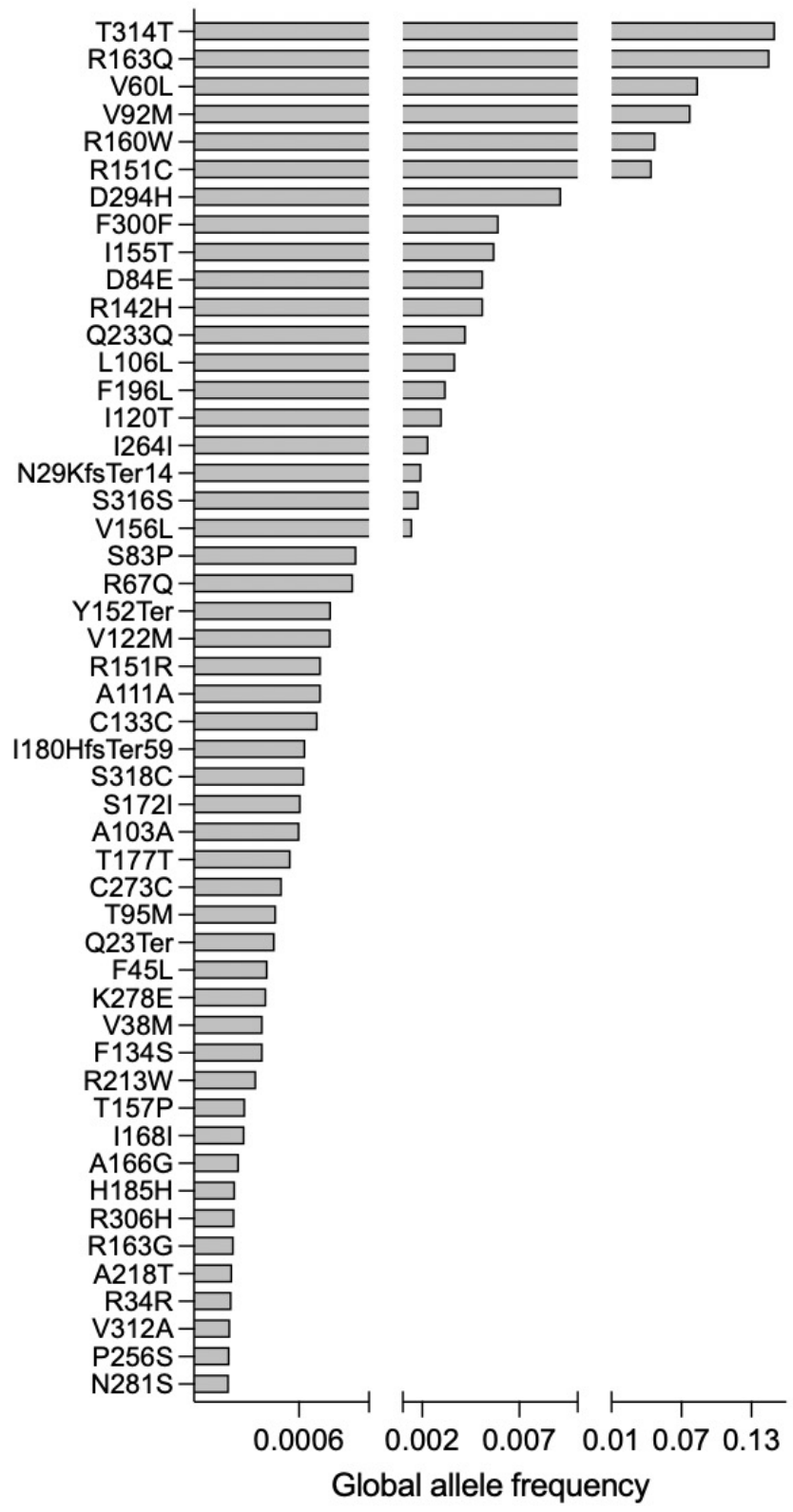

B

Variant ID	Nucleotide position	Nucleotide change	Amino acid position	Amino acid change	Global alele frequency	Homozygote (count)	Homozygote (% carriers)	Homocygote (% total)	Expected homozygote (% total)
rs885479	488	G>A	163	R>Q	1.46E-01	7912	24.5	5.74E+00	2.12E+00
rs2228478	942	A>G	314	T>T	1.50E-01	4830	13.0	3.45E+00	2.26E+00
rs3212371	*581	A>G	3 prime UTR	A>G	1.41E-01	1898	10.4	2.66E+00	1.98E+00
rs1805005	178	G>T	60	V>L	8.42E-02	1591	7.2	1.14E+00	7.08E-01
rs2228479	274	G>A	92	V>M	7.79E-02	1477	7.3	1.06E+00	6.07E-01
rs1805008	478	C>T	160	R>W	4.76E-02	487	3.9	3.58E-01	2.27E-01
rs1805007	451	C>T	151	R>C	4.48E-02	466	3.9	3.40E-01	2.01E-01
rs3212368	*12	G>A	3 prime UTR	G>A	1.46E-02	294	7.8	2.12E-01	2.13E-02
rs3212367	900	C>T	300	F>F	5.95E-03	48	3.0	3.42E-02	3.54E-03
rs1805009	880	G>C	294	D>H	9.16E-03	23	0.9	1.64E-02	8.39E-03
rs3212364	318	G>A	106	L>L	3.70E-03	21	2.1	1.51E-02	1.37E-03
rs3212366	586	T>C	196	F>L	3.22E-03	13	1.5	9.47E-03	1.03E-03
rs146544450	699	G>A	233	Q>Q	4.26E-03	9	0.8	6.47E-03	1.82E-03
rs33932559	359	T>C	120	I>T	3.01E-03	8	1.0	5.79E-03	9.05E-04
rs11547464	425	G>A	142	R>H	5.14E-03	7	0.5	5.09E-03	2.64E-03
rs1805006	252	C>A	84	D>E	5.14E-03	7	0.5	5.00E-03	2.64E-03
rs376670171	515	G>T	172	S>I	6.10E-04	5	3.0	3.61E-03	3.73E-05
rs368745976	333	G>A	111	A>A	7.25E-04	3	1.5	2.16E-03	5.26E-05
rs34090186	200	G>A	67	R>Q	9.10E-04	3	1.2	2.14E-03	8.28E-05
rs181269865	792	C>T	264	I>I	2.33E-03	3	0.5	2.14E-03	5.45E-04
rs143513600	*337	A>G	3 prime UTR	A>G	1.65E-03	1	0.5	1.67E-03	2.72E-04
rs201326893	456	C>A	152	Y*	7.84E-04	2	0.9	1.46E-03	6.15E-05
rs145781072	531	G>A	177	T>T	5.54E-04	2	1.3	1.45E-03	3.07E-05
rs34474212	247	T>C	83	S>P	9.28E-04	2	0.8	1.43E-03	8.62E-05
rs541142974	-32	dupC	5 prime UTR		3.76E-04	1	1.8	1.32E-03	1.42E-05
rs375789795	-13	C>T	5 prime UTR	C>T	4.45E-04	1	1.2	1.08E-03	1.98E-05
rs564161331	401	T>C	134	F>S	3.93E-04	1	1.1	8.18E-04	1.54E-05
rs762063106	949	T>C	317	W>R	4.43E-05	1	10.0	8.05E-04	1.96E-07
rs963182706	223	T>C	75	C>R	1.21E-05	1	50.0	8.04E-04	1.45E-08
rs773094322	854	C>G	285	A>G	4.41E-05	1	10.0	8.03E-04	1.95E-07
rs3212365	466	G>C	156	V>L	1.49E-03	1	0.2	7.32E-04	2.23E-04
rs1110400	464	T>C	155	I>T	5.72E-03	1	0.1	7.31E-04	3.28E-03
rs527673999	577	G>A	193	V>M	1.13E-04	1	3.3	7.28E-04	1.27E-06
rs201533137	67	C>T	23	Q*	4.62E-04	1	0.8	7.27E-04	2.13E-05
rs201489928	332	C>T	111	A>V	1.59E-04	1	2.3	7.22E-04	2.52E-06
rs151318945	948	C>T	316	S>S	1.83E-03	1	0.2	7.14E-04	3.33E-04
rs141177570	842	A>G	281	N>S	2.00E-04	1	1.8	7.13E-04	3.99E-06

Figure S1. Frequency of MC1R variants and homozygotes analysis. Data was obtained from gnomAD v2.1.1 for Transcript ENST00000555147.1, August 2023. **A)** Global allele frequency of the 50 most frequent variants at *MC1R* . **B)** Variants for which homozygotes have been identified, ranked by homozygotes count (blue). The following values are shown: percentage of carriers which are in homozygous state, frequency of homozygotes respect to the total population; expected frequency of homozygotes based on Hardy-Weinberg principle calculated as 100 x (global allele frequency)^2.

Fig. S2

Variant ID	Nt position	Nt change	Amino acid position	Amino acid change	Global alele frequency	European non-Finnish	Finnish	African/ American	Latino Admixed	South Asia	East Asia	Jewish	Other
rs776654211	52	C > G	18	P > A	1.85E-05	4.08E-05	0.00E+00	0.00E+00	0.00E+00	0.00E+00	0.00E+00	0.00E+00	0.00E+00
rs200050206	112	G > A	38	V > M	3.94E-04	8.00E-04	4.00E-05	2.49E-04	0.00E+00	0.00E+00	0.00E+00	0.00E+00	1.41E-04
rs1805005	178	G > T	60	V > L	8.42E-02	1.20E-01	5.70E-02	1.91E-02	6.37E-02	1.16E-02	1.54E-04	2.88E-01	1.09E-01
rs34090186	200	G > A	67	R > Q	9.10E-04	1.09E-04	0.00E+00	8.27E-05	0.00E+00	1.05E-03	1.04E-02	0.00E+00	4.21E-04
rs749221540	227	T > A	76	F > Y	4.02E-06	8.88E-06	0.00E+00	0.00E+00	0.00E+00	0.00E+00	0.00E+00	0.00E+00	0.00E+00
rs772286882	241	G > C	81	A > P	6.43E-05	5.47E-05	0.00E+00	0.00E+00	0.00E+00	0.00E+00	0.00E+00	1.07E-03	0.00E+00
rs34474212	247	T > C	83	S > P	9.28E-04	3.91E-04	0.00E+00	0.00E+00	1.98E-03	0.00E+00	0.00E+00	1.21E-02	2.10E-03
rs1805006	252	C > A	84	D > E	5.14E-03	9.55E-03	1.88E-03	1.73E-03	2.01E-03	0.00E+00	0.00E+00	1.94E-03	5.32E-03
rs34540312	265	G > A	89	G > R	1.00E-04	0.00E+00	0.00E+00	1.03E-03	5.66E-05	0.00E+00	0.00E+00	0.00E+00	1.40E-04
rs2228479	274	G > A	92	V > M	7.79E-02	8.40E-02	7.30E-02	1.61E-02	4.54E-02	2.41E-02	2.77E-01	6.06E-02	6.77E-02
rs34158934	284	C > T	95	T > M	4.69E-04	5.71E-04	0.00E+00	4.96E-04	1.02E-03	9.81E-05	0.00E+00	0.00E+00	9.82E-04
rs1442510320	289	G > A	97	V > I	1.21E-05	8.90E-06	0.00E+00	0.00E+00	0.00E+00	3.27E-05	5.57E-05	0.00E+00	0.00E+00
rs201192930	364	G > A	122	V > M	7.82E-04	4.77E-04	0.00E+00	2.04E-04	1.19E-03	0.00E+00	5.13E-05	8.61E-03	2.53E-03
rs11547464	425	G > A	142	R > H	5.14E-03	6.84E-03	1.36E-03	9.71E-04	6.76E-03	1.64E-04	5.13E-05	1.67E-02	1.01E-02
rs374423188	445	G > A	149	A > T	6.58E-05	8.03E-05	0.00E+00	3.15E-04	5.80E-05	0.00E+00	0.00E+00	0.00E+00	0.00E+00
rs1805007	451	C > T	151	R > C	4.48E-02	7.18E-02	7.05E-02	1.36E-02	9.57E-03	3.63E-03	6.67E-04	6.50E-02	4.25E-02
rs1110400	464	T > C	155	I > T	5.72E-03	9.29E-03	1.85E-03	2.26E-03	4.19E-03	4.58E-04	0.00E+00	8.55E-03	6.34E-03
rs1805008	478	C > T	160	R > W	4.76E-02	7.97E-02	6.56E-02	1.27E-02	8.19E-03	5.24E-03	5.14E-05	5.74E-02	4.24E-02
rs885479	488	G > A	163	R > Q	1.46E-01	4.91E-02	2.24E-01	1.73E-02	3.95E-01	3.75E-02	6.38E-01	3.45E-02	1.15E-01
rs762183931	520	G > A	174	V > I	8.15E-06	0.00E+00	0.00E+00	0.00E+00	0.00E+00	3.27E-05	0.00E+00	0.00E+00	1.66E-04
rs200000734	637	C > T	213	R > W	3.56E-04	5.06E-04	4.61E-05	5.46E-04	4.54E-04	9.82E-05	0.00E+00	0.00E+00	1.43E-04
rs200965363	652	G > A	218	A > T	2.19E-04	3.52E-04	0.00E+00	2.09E-04	2.84E-05	2.62E-04	5.15E-05	0.00E+00	1.42E-04
rs368714912	689	C > T	230	P > L	5.77E-05	6.34E-05	2.48E-04	0.00E+00	0.00E+00	6.54E-05	0.00E+00	0.00E+00	0.00E+00
rs767276470	755	T > A	252	L > H	NA	NA	NA	NA	NA	NA	NA	NA	NA
rs201171524	832	A > G	278	K > E	4.13E-04	4.59E-04	3.60E-04	8.27E-05	4.24E-04	9.15E-04	0.00E+00	2.90E-04	0.00E+00
rs202197434	837	C > A	279	N > K	9.62E-05	2.02E-04	0.00E+00	0.00E+00	2.83E-05	0.00E+00	0.00E+00	0.00E+00	0.00E+00
rs1805009	880	G > C	294	D > H	9.16E-03	1.62E-02	2.12E-03	3.40E-03	7.24E-03	0.00E+00	0.00E+00	1.64E-03	1.09E-02
rs752932359	896	C > T	299	A > V	8.03E-06	0.00E+00	0.00E+00	6.48E-05	0.00E+00	0.00E+00	0.00E+00	0.00E+00	1.65E-04
rs368507952	917	G > A	306	R > H	2.32E-04	2.18E-04	0.00E+00	1.24E-04	0.00E+00	1.05E-03	0.00E+00	0.00E+00	2.80E-04
rs761041641	943	T > C	315	C > R	1.78E-05	1.56E-05	0.00E+00	0.00E+00	8.49E-05	0.00E+00	0.00E+00	0.00E+00	0.00E+00

Figure S2. Variant information for the selected 30 variants in this study. Data was obtained from gnomAD v2.1.1 for Transcript ENST00000555147.1, August 2023. Nucleotide (Nt) and amino acid position and change are shown. Global allele frequency as well as frequency for each individual population included in gnomAD database are shown. This data corresponds to the diagram shown in Figure 1D. NA indicates data not available.

Fig. S3

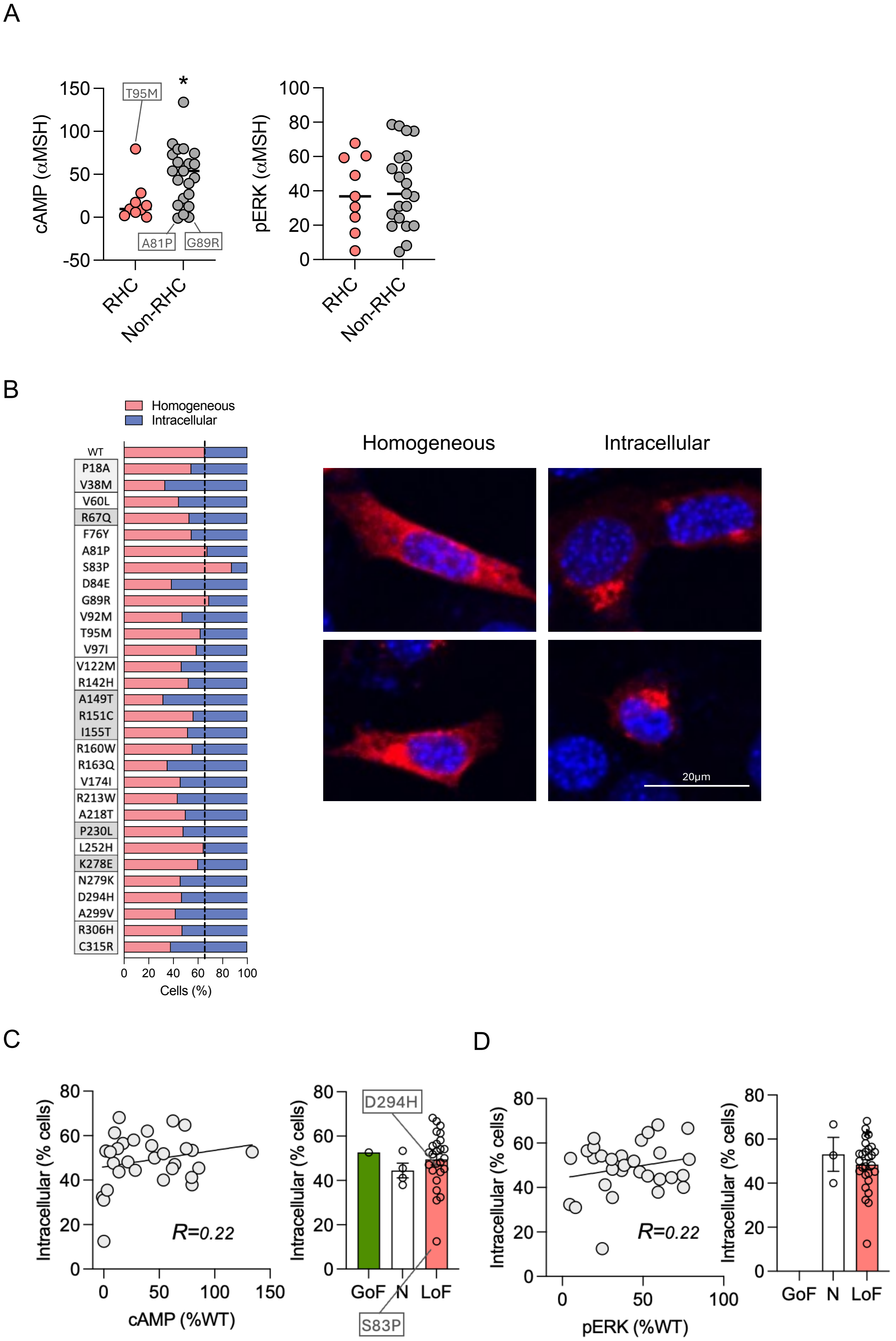

A
cAMP (αMSH)
150
100
50
0
-50
T95M
*
A81P
G89R
RHC
Non-RHC
pERK (αMSH)
100
80
60
40
20
0
RHC
Non-RHC
B
Homogeneous
Intracellular
WT
P18A
V38M
V60L
R67Q
F76Y
A81P
S83P
D84E
G89R
V92M
T95M
V97I
V122M
R142H
A149T
R151C
I155T
R160W
R163Q
V174I
R213W
A218T
P230L
L252H
K278E
N279K
D294H
A299V
R306H
C315R
0 20 40 60 80 100
Cells (%)
Homogeneous
Intracellular
20μm
C
Intracellular (% cells)
80
60
40
20
0
0 50 100 150
cAMP (%WT)
R=0.22
D294H
GoF N LoF
S83P
D
Intracellular (% cells)
80
60
40
20
0
0 50 100
pERK (%WT)
R=0.22
GoF N LoF

Figure S3. cAMP and pERK responses association with red hair colour variants, receptor expression and intrinsic activity. **A)** cAMP and pERK pathways measured in HEK293 cells transfected with each of the *MC1R* variants upon stimulation with 10μM αMSH for 15 and 5 minutes respectively. Data is represented according to variants being classified as RHC or not. Data represent mean±SEM and analysed by t-test **B-D)** NIH-3T3 cells were transfected with each of the *MC1R* variants and receptor expression detected by immunofluorescence using an anti-Myc tag antibody. Receptor expression using a confocal microscope was detected mostly following two patterns: homogeneously distributed through the cell, or intracellularly retained, likely in the Golgi or endoplasmic reticulum. Percentage of MC_1^+ cells displaying each of the expression patterns, and representative images are shown in **B**). For each variant, the proportion of cells displaying intracellularly retained expression of the receptor were quantified and represented against cAMP (**C**) or pERK (**D**) response to αMSH. Data represent mean±SEM, or individual values in the scatter plot, and analysed by Pearson correlation test or one-way ANOVA. *$p<0.05$.

Fig. S4

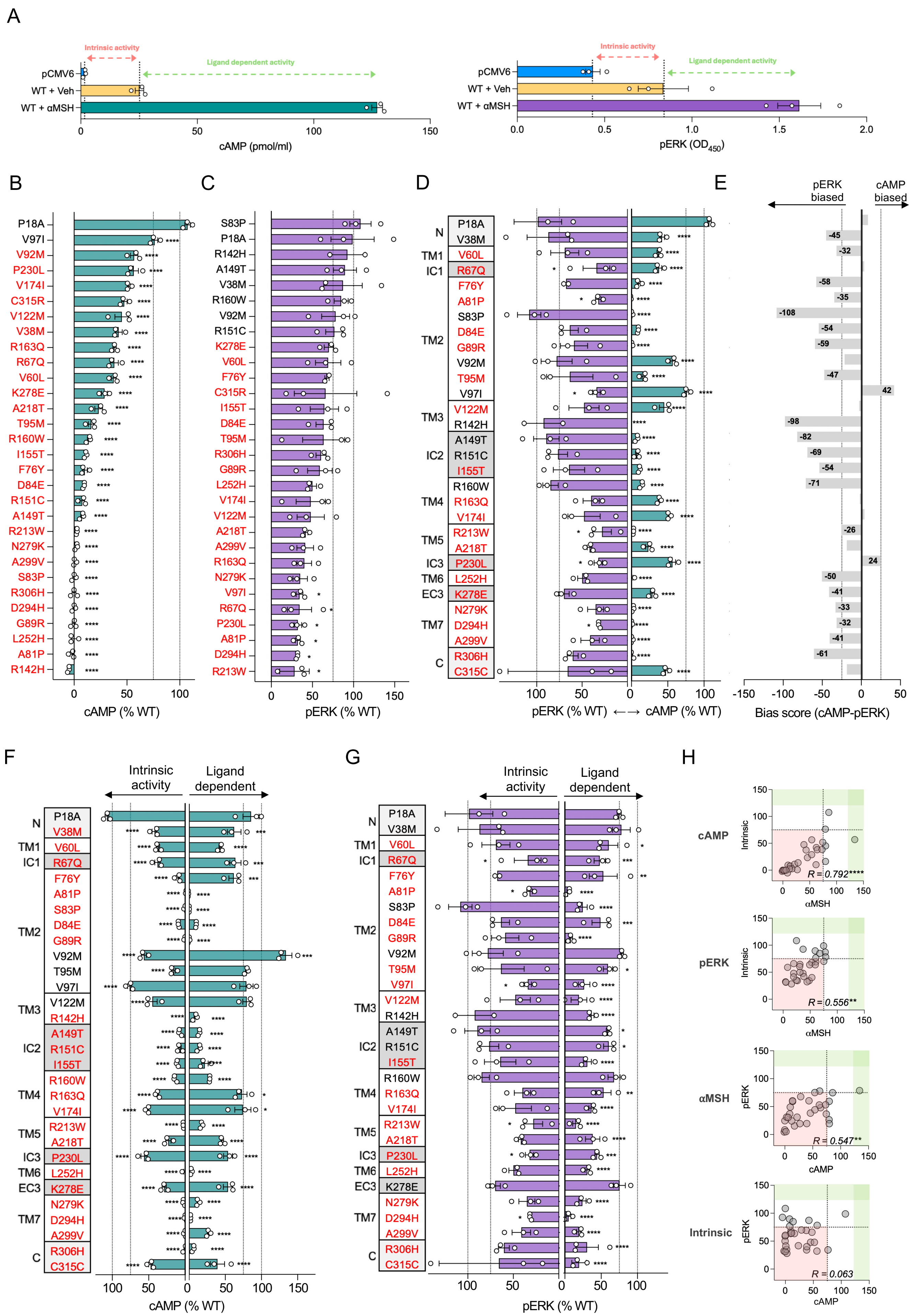

A
Intrinsic activity
Ligand dependent activity
pCMV6
WT + Veh
WT + αMSH
cAMP (pmol/ml)
pERK (OD450)
B
cAMP (% WT)
C
pERK (% WT)
D
pERK (% WT) ←→ cAMP (% WT)
E
pERK biased
cAMP biased
Bias score (cAMP-pERK)
F
Intrinsic activity
Ligand dependent
cAMP (% WT)
G
Intrinsic activity
Ligand dependent
pERK (% WT)
H
cAMP
pERK
αMSH
Intrinsic
R = 0.792****
R = 0.556**
R = 0.547**
R = 0.063

Figure S4. Effect of *MC1R* variants in the ligand-independent intrinsic signalling response. cAMP and pERK pathways measured in HEK293 transfected with the empty vector (pCMV6) or the wild type form of *MC1R* (WT), upon treatment for 15 minutes (cAMP) or 5 minutes (pERK). **A)** Data depicting the existence of intrinsic activity (WT + Veh) compared with ligand-dependent activity (αMSH, 10μM), using empty-vector (pCMV6) transfected cells as control. Data is presented as pmol/ml of cAMP and optic density at 450nm (pERK). **B)** cAMP responses ranked by response as % of WT (LoF in red). **C)** pERK responses ranked by response as % of WT (LoF in red). **D)** cAMP and pERK responses (from B and C) ranked by amino acid order (LoF in both pathways in red). **E)** Biased score calculated by subtracting pERK from cAMP responses. Values near zero indicate lack of bias. **F)** Comparison of cAMP responses in vehicle treated cells (intrinsic activity, shown in B) *vs*. αMSH treated cells (from Fig. 1E) **G)** Comparison of pERK responses in vehicle treated cells (intrinsic activity, shown in C) *vs*. αMSH treated cells (from Fig. 1F). **H)** Correlation between cAMP, pERK , intrinsic and αMSH-derived responses. Data represent mean±SEM of n=3 and analysed by one-way ANOVA with Dunnett's multiple comparison test *vs*. WT (B-D, F,G), or Pearson correlation test (H); *$p<0.05$, **$p<0.01$, ***$p<0.001$, ****$p<0.0001$.

Fig. S5

A

MC1R cAMP	AM	SIFT	Polyphen	CADD	REVEL	MetaLR	Mut As
**PPV**	100	100	100	0	100	100	95
**NPV**	19	42	26	17	19	19	40
**Sensitivity**	12	72	44	0	12	12	76
**Specificity**	100	100	100	100	100	100	80
**Accuracy**	27	77	53	17	27	27	77

B

MC1R pERK	AM	SIFT	Polyphen	CADD	REVEL	MetaLR	Mut As
**PPV**	100	94	100	0	100	100	95
**NPV**	11	17	16	10	11	11	20
**Sensitivity**	11	63	41	0	11	11	70
**Specificity**	100	67	100	100	100	100	67
**Accuracy**	20	63	47	10	20	20	70

C

MC1R RHC	AM	SIFT	Polyphen	CADD	REVEL	MetaLR	Mut As
**PPV**	33	44	45	0	100	100	45
**NPV**	70	92	79	70	78	78	100
**Sensitivity**	11	89	56	0	33	33	100
**Specificity**	90	52	71	100	100	100	48
**Accuracy**	67	63	67	70	80	80	63

D

MC4R	**AM**		**SIFT**		**Polyphen**		**CADD**		**REVEL**		**MetaLR**		**Mut As**	
cAMP	LoF	L+GoF	LoF	L+GoF	LoF	L+GoF	LoF	L+GoF	LoF	L+GoF	LoF	L+GoF	LoF	L+GoF
**PPV**	87	93	59	66	67	71	0	0	76	81	75	75	64	76
**NPV**	81	58	92	50	80	50	56	39	90	60	59	41	88	63
**Sensitivity**	72	56	94	76	78	60	0	0	89	68	17	12	89	76
**Specificity**	91	94	48	38	70	63	100	100	78	75	96	94	61	63
**Accuracy**	83	71	68	61	73	61	56	39	83	71	61	44	73	71

E

MC4R	**AM**		**SIFT**		**Polyphen**		**CADD**		**REVEL**		**MetaLR**		**Mut As**	
βArrestin	LoF	L+GoF	LoF	L+GoF	LoF	L+GoF	LoF	L+GoF	LoF	L+GoF	LoF	L+GoF	LoF	L+GoF
**PPV**	87	87	83	86	95	100	0	0	90	90	100	100	84	88
**NPV**	31	12	42	8	45	19	24	12	40	15	71	16	38	13
**Sensitivity**	42	36	77	69	65	42	0	0	61	53	87	11	68	61
**Specificity**	80	60	50	20	90	100	100	100	80	60	100	100	60	40
**Accuracy**	51	39	71	63	71	49	24	12	66	54	90	24	66	59

Figure S5. Test characteristics for the pathogenicity predictors. Positive predicted value (PPV), negative predictive value (NPV), AlphaMissense (AM), Mut As (Mutation Assessor). Loss of function (LoF), gain of function (GoF). Test characteristics for MC1R on cAMP **(A)** and pERK **(B)** and association with red hair (RHC) **(C)**. Test characteristics for *MC4R* (data obtained from Lotta *et al*, Cell 2019 Apr 18;177(3):597-607.e9.) on cAMP **(D)** and β-arrestin **(E)**. *PPV*: probability that a variant testing pathogenic is truly pathogenic, high values indicate low chances of false positives; *NPP*: probability that a variant testing benign is truly benign, high values indicate low chances of false negatives; *Sensitivity*: percentage of true positives; *Specificity*: percentage of true negatives; *Accuracy*: proportion of cases (both benign and pathogenic) correctly identified.

Fig. S6

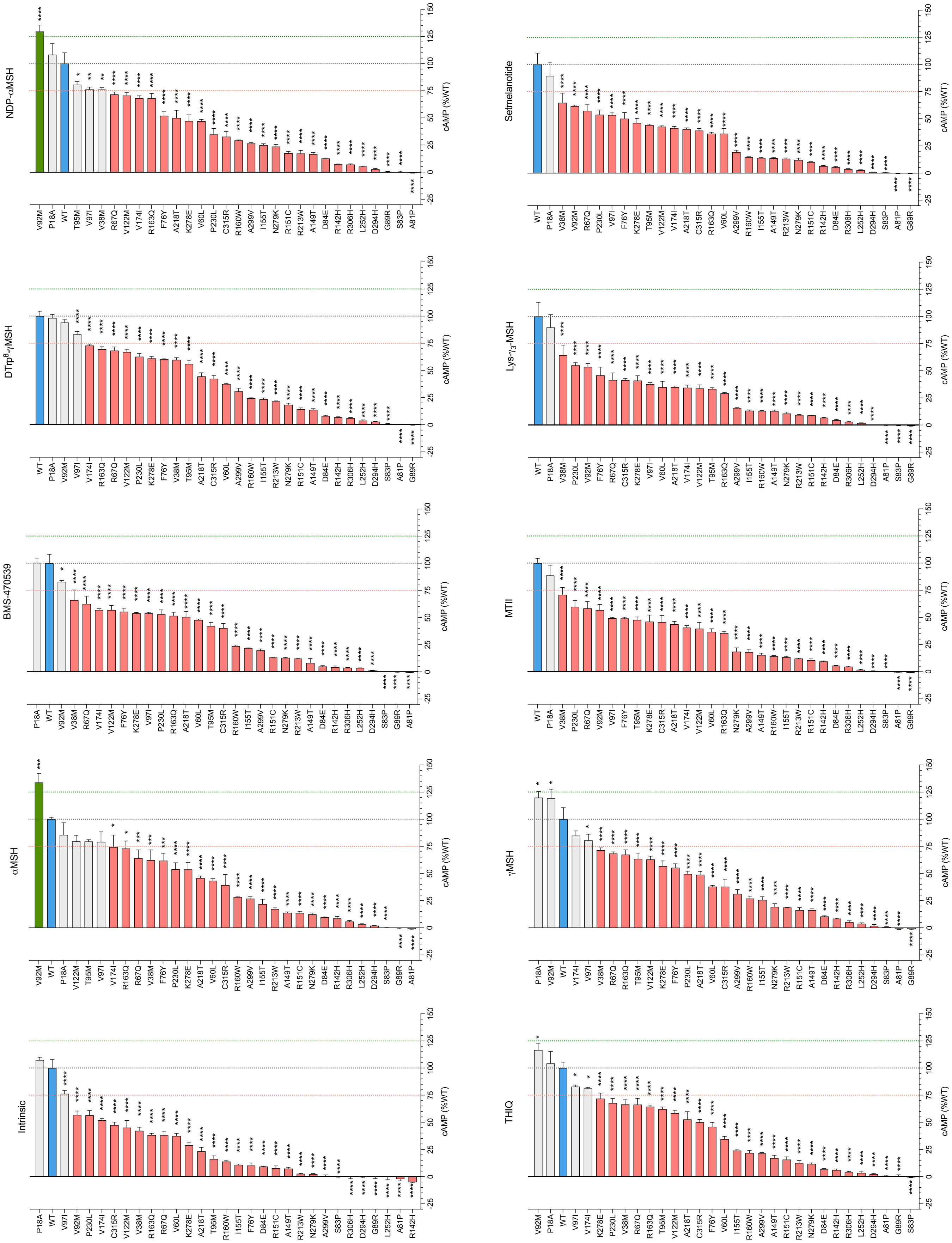

NDP-αMSH
Setmelanotide
DTrp⁸-γMSH
Lys-γ₃-MSH
BMS-470539
MTII
αMSH
γMSH
Intrinsic
THIQ
cAMP (%WT)

Figure S6. cAMP responses by melanocortin compounds on *MC1R* variants. Transfected HEK293 were stimulated with vehicle (intrinsic), αMSH, BMS-470539, DTrp^{8}-γMSH, NDP-αMSH, THIQ, γMSH, MTII, Lys-γ_3MSH or setmelanotide for 15 minutes. Data is presented as percentage of response obtained in cells transfected with the wild type (WT) form of *MC1R* (blue bar*)*. Values ≥25% or ≤25% vs. wild type were considered GoF (green bars) or LoF (red vars) respectively. Data is ranked by response. Data represent mean±SEM of n=3, one-way ANOVA with Dunnett's multiple comparison test *vs*. WT; *p<0.05, **p<0.01, ***p<0.001, ****p<0.0001.

Fig. S7

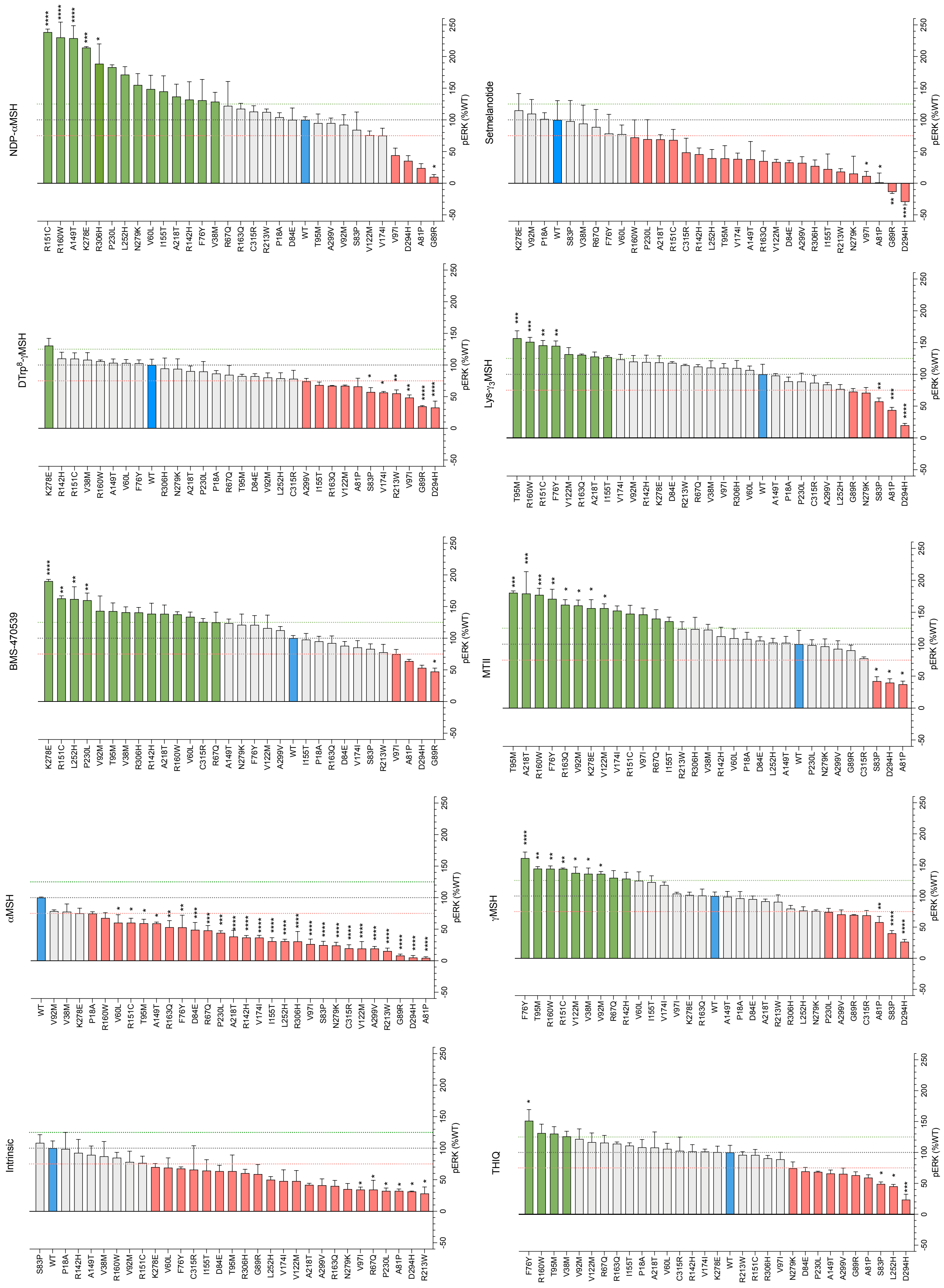
NDP-αMSH
Setmelanotide
DTrp8-γMSH
Lys-γ3MSH
BMS-470539
MTII
αMSH
γMSH
Intrinsic
THIQ
pERK (%WT)

Figure S7. pERK responses by melanocortin compounds on *MC1R* variants. Transfected HEK293 were stimulated with vehicle (intrinsic), αMSH, BMS-470539, DTrp^8-γMSH, NDP-αMSH, THIQ, γMSH, MTII, Lys-γ_3MSH or setmelanotide for 5 minutes. Data is presented as percentage of response obtained in cells transfected with the wild type (WT) form of *MC1R* (blue bar). Values ≥25% or ≤25% vs. wild type were considered GoF (green bars) or LoF (red vars) respectively. Data is ranked by response. Data represent mean±SEM of n=3, one-way ANOVA with Dunnett's multiple comparison test *vs*. WT; *p<0.05, **p<0.01, ***p<0.001, ****p<0.0001.

Fig. S8

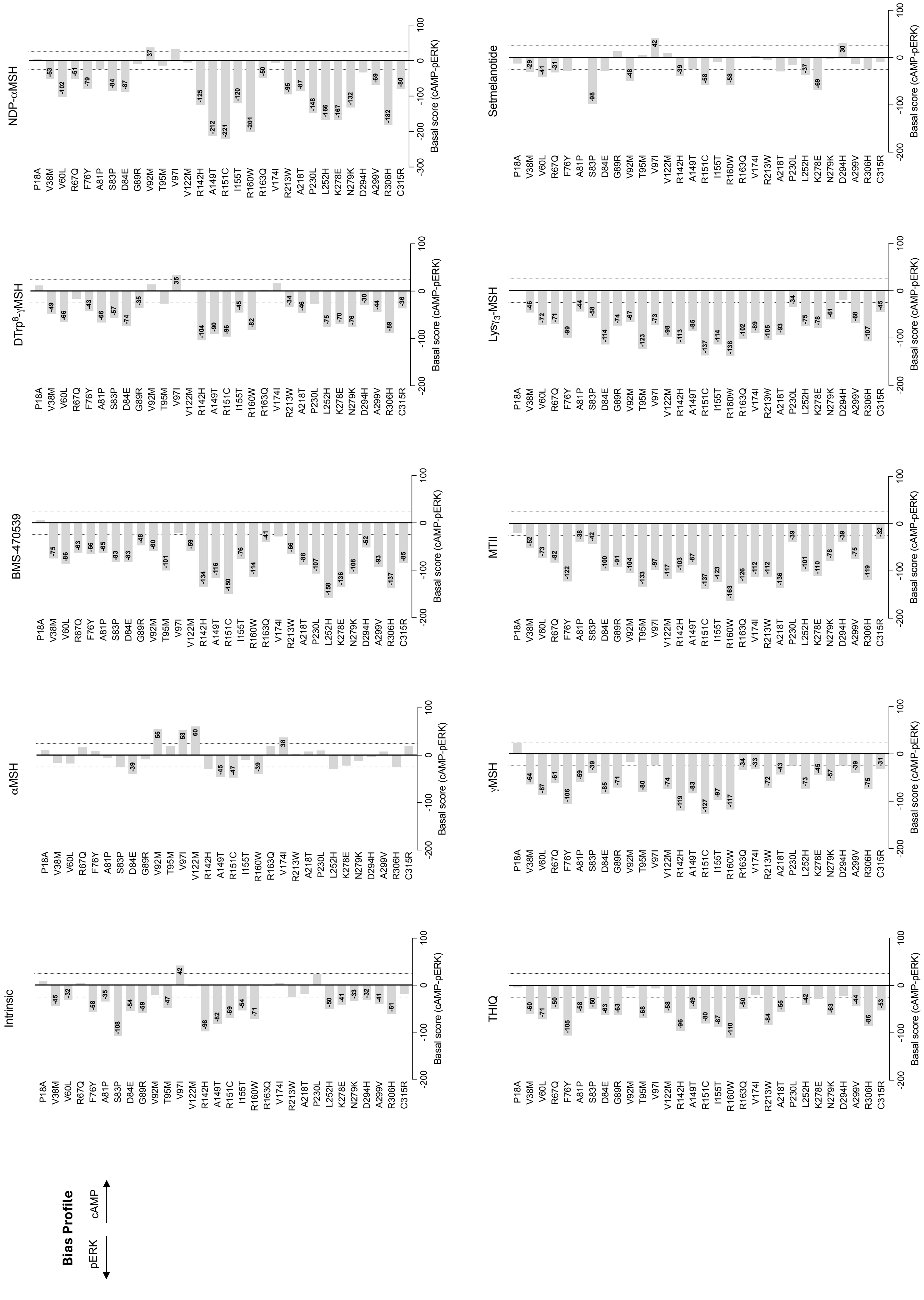

NDP-αMSH
Setmelanotide
DTrp8-γMSH
Lysγ3-MSH
BMS-470539
MTII
αMSH
γMSH
Intrinsic
THIQ
Basal score (cAMP-pERK)
Bias Profile
pERK
cAMP

Figure S8. Bias score for all melanocortin compounds at each *MC1R* variant. Values on cAMP and pERK represented in Supplementary Figure 6 and 7 were used to calculate the bias score by subtracting the pERK values from the cAMP values. Values near zero indicate lack of bias. Variants are ranked by amino acid position.

Fig. S9

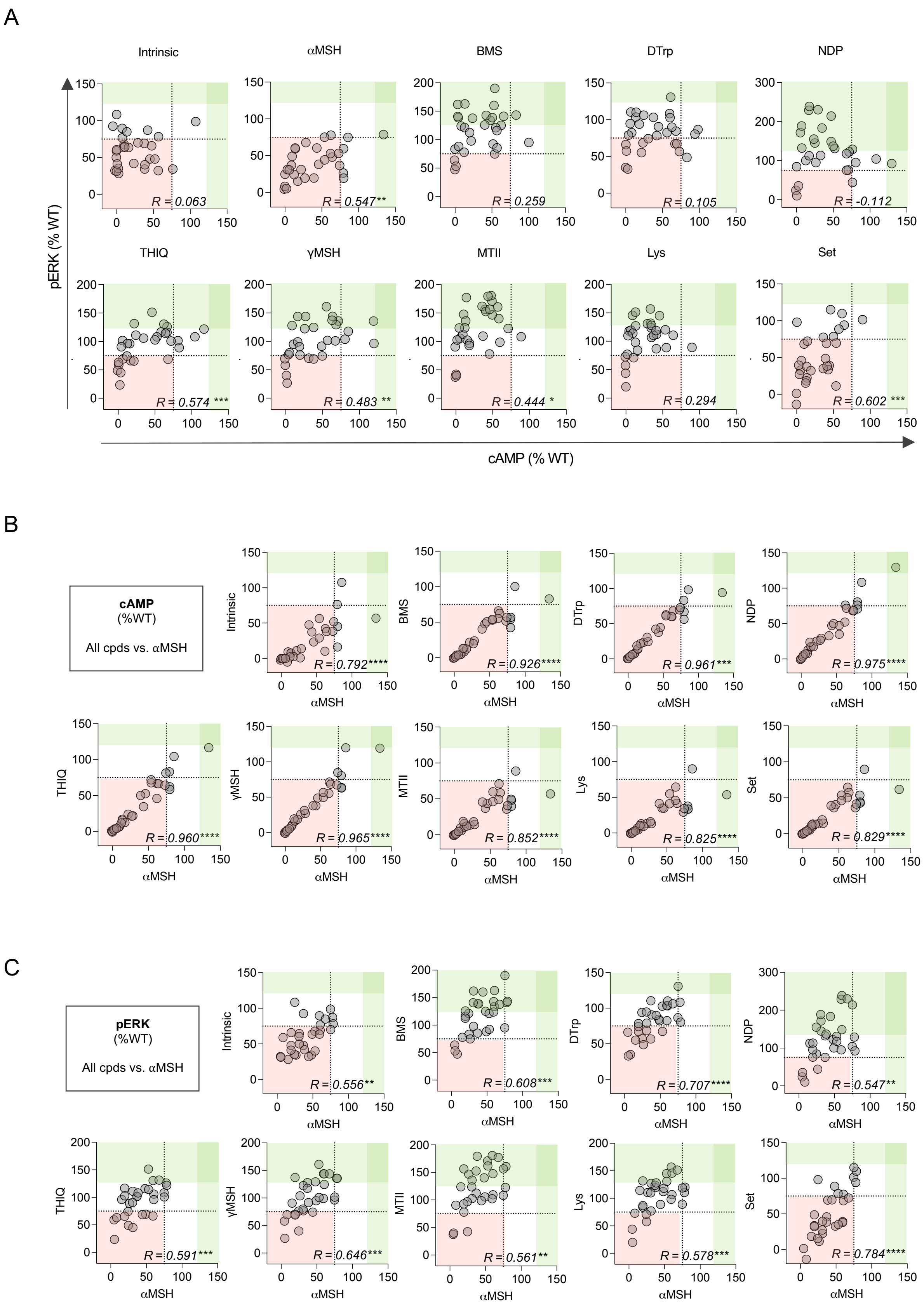

A
Intrinsic
αMSH
BMS
DTrp
NDP
R = 0.063
R = 0.547**
R = 0.259
R = 0.105
R = -0.112
THIQ
γMSH
MTII
Lys
Set
R = 0.574 ***
R = 0.483 **
R = 0.444 *
R = 0.294
R = 0.602 ***
pERK (% WT)
cAMP (% WT)
B
cAMP
(%WT)
All cpds vs. αMSH
Intrinsic
BMS
DTrp
NDP
R = 0.792****
R = 0.926****
R = 0.961***
R = 0.975****
THIQ
γMSH
MTII
Lys
Set
R = 0.960****
R = 0.965****
R = 0.852****
R = 0.825****
R = 0.829****
αMSH
C
pERK
(%WT)
All cpds vs. αMSH
Intrinsic
BMS
DTrp
NDP
R = 0.556**
R = 0.608***
R = 0.707****
R = 0.547**
THIQ
γMSH
MTII
Lys
Set
R = 0.591***
R = 0.646***
R = 0.561**
R = 0.578***
R = 0.784****
αMSH

Figure S9. Associations between melanocortin compounds and responses at each *MC1R* variant. Values on cAMP and pERK responses represented in Supplementary Figure 6 and 7 were used to calculate the correlation between the different activities and compounds. Each data point represents one of the 30 variants included in this study. **A)** Correlation analysis between cAMP and pERK responses for each of the compounds, including vehicle (intrinsic). **B)** Correlation of cAMP responses between αMSH and all other compounds, including vehicle (intrinsic). **C)** Correlation of pERK responses between αMSH and all other compounds, including vehicle (intrinsic). Data was analysed using Person correlation test; *$p<0.05$, **$p<0.01$, ***$p<0.001$, ****$p<0.0001$.

Fig. S10

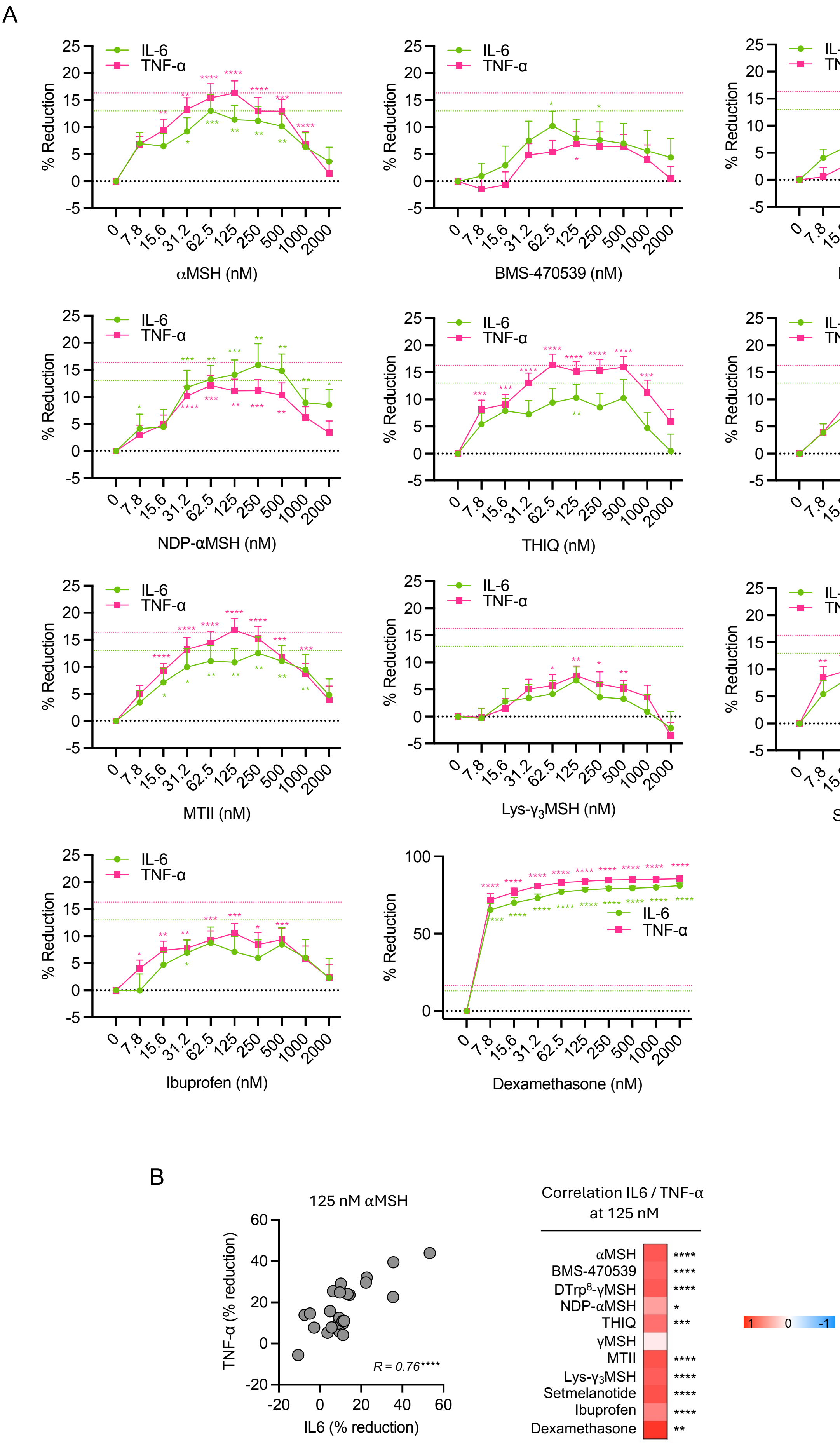
A
IL-6
TNF-α
% Reduction
αMSH (nM)
BMS-470539 (nM)
DTrp^{8}-γMSH (nM)
NDP-αMSH (nM)
THIQ (nM)
γMSH (nM)
MTII (nM)
Lys-γ$_{3}$MSH (nM)
Setmelanotide (nM)
Ibuprofen (nM)
Dexamethasone (nM)
B
125 nM αMSH
TNF-α (% reduction)
IL6 (% reduction)
R = 0.76****
Correlation IL6 / TNF-α at 125 nM
αMSH ****
BMS-470539 ****
DTrp^{8}-γMSH ****
NDP-αMSH *
THIQ ***
γMSH
MTII ****
Lys-γ$_{3}$MSH ****
Setmelanotide ****
Ibuprofen ****
Dexamethasone **
1
0
-1

Figure S10. Efficacy of melanocortin compounds on the whole blood assay. Diluted whole blood was treated for 30min with melanocortin drugs (7.8-2000 nM) before being stimulated with 1 µg/ml LPS. Samples were collected 3 h later and cytokine release in supernatants analysed by ELISA. Vehicle (PBS), dexamethasone and ibuprofen were used as controls. Samples were analysed in duplicate. Mean intra-assay coefficients of variation (CVs) were 5.93% for IL6 and 5.36% for TNF-α. Data for all donors and compounds is normalised as % of reduction in cytokine content with respect to vehicle control. Vehicle values prior normalisation were 690±72 pg/ml for IL6 and 872±81 pg/ml for TNF-α. **A)** Reduction on IL6 (green) and TNF-α (pink) release by αMSH, BMS-470539, DTrp^{8}-γMSH, NDP-αMSH, THIQ, γMSH, MTII, Lys$_{3}$-γMSH, setmelanotide, ibuprofen and dexamethasone. **B)** The association between IL6 and TNF-α responses are shown for all compounds. Data is expressed as mean±SEM of n=25 donors, each one tested in duplicates. Concentration-response curves were analysed by two-way ANOVA, using non-transformed original values (i.e. as pg/ml). Association analysis was performed by Pearson correlation test; ***p<0.001, ****p<0.0001.

Fig. S11

Donor	IL6 (% reduction)										
	αMSH	BMS	DTrp	NDP	THIQ	γMSH	MTII	Lys	Set	Ibu	Dex
1	42.20	38.29	42.27	38.07	34.22	26.12	48.87	35.54	40.46	15.42	125.51
2	5.54	17.62	-0.03	19.53	-0.11	8.99	3.70	9.42	2.58	0.40	88.83
3	-9.05	26.05	21.42	25.29	1.75	-6.98	17.37	11.43	-0.65	-5.62	79.45
4	5.27	17.67	15.48	11.93	6.09	3.01	4.24	-14.36	18.51	-7.87	81.34
5	0.62	8.77	14.78	7.49	-13.15	2.88	0.77	-16.40	-13.95	-2.85	82.30
6	8.72	-1.89	23.24	3.28	11.46	2.89	23.09	-4.71	9.27	7.25	69.65
7	6.40	13.58	10.50	10.90	4.20	1.98	15.49	-12.42	5.44	0.84	83.59
8	32.18	36.86	15.77	35.66	17.28	17.08	31.35	0.80	19.05	8.60	75.61
9	-5.97	6.24	2.59	-1.91	7.01	2.61	5.03	3.93	8.83	8.18	80.59
10	7.98	11.73	7.42	12.55	-2.72	8.43	8.19	2.48	-5.12	2.26	80.56
11	-2.39	4.49	7.85	7.01	2.27	2.01	10.13	-4.66	-3.94	5.99	64.24
12	-2.70	5.75	6.48	9.81	-0.12	1.20	4.17	2.97	-1.00	-0.22	77.99
13	23.53	19.42	22.41	28.78	39.42	5.02	28.05	27.46	35.17	38.62	85.19
14	10.07	17.45	22.96	34.06	16.13	4.36	15.56	26.62	14.17	0.59	69.48
15	7.88	4.69	-4.94	10.43	5.38	14.58	6.00	9.37	8.32	9.11	73.16
16	13.49	-11.45	1.68	0.53	3.35	18.54	15.21	3.98	4.40	-5.52	73.34
17	16.36	20.98	14.39	22.35	6.10	15.57	3.85	1.99	11.27	-2.70	85.34
18	5.84	4.09	2.43	2.41	0.87	2.66	8.78	0.48	2.06	2.13	81.89
19	1.04	8.15	6.14	9.31	-0.30	6.03	-1.83	3.37	0.98	37.35	78.86
20	6.08	7.61	7.06	5.71	-10.60	3.71	5.80	1.82	-0.97	0.96	85.78
21	3.50	-37.77	-13.65	-29.04	14.94	-13.02	-29.44	-5.71	14.66	0.49	57.28
22	24.79	6.78	6.93	7.03	16.85	22.39	4.32	18.26	17.95	14.54	77.44
23	16.40	-16.29	6.79	4.77	10.25	7.06	6.34	2.44	11.09	4.16	54.75
24	21.01	-18.58	10.77	9.49	21.89	15.05	14.59	7.78	19.43	9.63	75.99
25	10.47	-3.93	4.58	23.40	25.07	8.74	13.76	-12.38	10.97	33.51	67.65

Donor	TNF-α (% reduction)										
	αMSH	BMS	DTrp	NDP	THIQ	γMSH	MTII	Lys	Set	Ibu	Dex
1	39.27	36.93	19.68	31.43	30.81	11.74	46.45	18.63	34.12	20.97	123.83
2	9.53	14.91	5.14	9.89	1.26	7.76	13.24	7.76	1.41	-2.57	84.60
3	11.76	16.05	21.02	20.32	8.58	13.67	21.86	11.55	14.28	2.23	84.04
4	19.29	10.31	5.98	10.67	16.22	12.07	8.60	-5.86	20.16	-0.88	85.14
5	3.93	12.14	-7.35	6.41	6.50	-0.81	2.97	-3.70	7.23	7.13	83.35
6	14.30	13.46	13.36	19.12	13.50	12.49	21.01	-1.65	10.20	5.95	83.22
7	14.23	7.59	8.40	6.22	14.72	1.88	14.94	-7.82	16.25	6.92	86.57
8	37.25	24.72	20.27	22.30	31.68	26.31	35.22	12.20	31.11	18.55	79.24
9	-7.04	1.47	-1.86	-3.16	16.49	-6.44	11.32	9.24	18.69	12.52	84.59
10	12.26	-3.83	0.19	3.89	10.02	8.74	10.48	-5.47	4.33	0.37	83.22
11	5.37	-0.49	3.04	-0.50	-2.54	4.76	14.95	0.05	-8.79	6.40	77.56
12	9.18	8.20	4.47	12.58	6.05	4.48	9.66	12.52	7.07	6.47	80.84
13	31.82	6.27	25.26	16.20	36.53	36.20	25.41	20.08	28.00	34.81	83.88
14	8.54	0.16	16.26	13.55	21.44	4.33	20.41	10.07	17.09	5.91	72.25
15	9.03	-5.54	-11.83	-1.54	10.60	2.18	8.19	8.88	13.12	1.32	85.61
16	12.49	-6.91	2.76	2.35	10.14	11.03	13.56	9.98	13.86	8.12	86.49
17	21.06	11.16	10.57	13.28	13.10	17.79	9.70	0.33	15.90	0.81	84.28
18	-0.14	1.63	-2.89	-3.00	14.61	-7.30	-2.99	10.89	14.93	5.09	87.69
19	9.04	-5.79	8.42	-2.04	4.67	9.05	6.03	9.36	5.59	25.26	82.26
20	10.19	10.47	15.01	8.70	17.17	12.04	7.10	10.85	9.67	19.63	89.52
21	3.14	0.28	2.43	12.32	15.31	7.83	3.58	-0.37	16.09	4.79	70.44
22	16.74	1.44	9.62	2.96	12.30	14.77	14.66	8.12	15.39	12.12	83.13
23	20.67	-14.44	2.39	6.12	15.59	11.35	9.04	-2.22	11.33	4.33	70.96
24	17.62	-7.47	5.94	15.78	10.79	6.99	16.69	-4.34	12.72	4.33	80.25
25	2.54	-10.01	-2.39	6.90	11.49	0.53	2.52	-4.02	6.08	3.67	77.38

Responder: ≥10% response
Non responder: <10% response

Figure S11. Summary efficacy of melanocortin compounds on the whole blood assay. Diluted whole blood was treated for 30min with melanocortin drugs (7.8-2000 nM) before being stimulated with 1 µg/ml LPS. Samples were collected 3 h later and cytokine release in supernatants analysed by ELISA. Vehicle (PBS), dexamethasone and ibuprofen were used as controls. Data for all donors and compounds is normalised as % of reduction in cytokine content with respect to vehicle control. Reduction on IL6 (green) and TNF-α (pink) release by αMSH, BMS-470539, DTrp^{8}-γMSH, NDP-αMSH, THIQ, γMSH, MTII, Lys-γ_3MSH, setmelanotide, ibuprofen and dexamethasone. Data is expressed as the average % reduction on each cytokine from 15.6-1000 nM obtained for each compound. Values ≥10% are highlighted in grey and considered responders.

Fig. S12

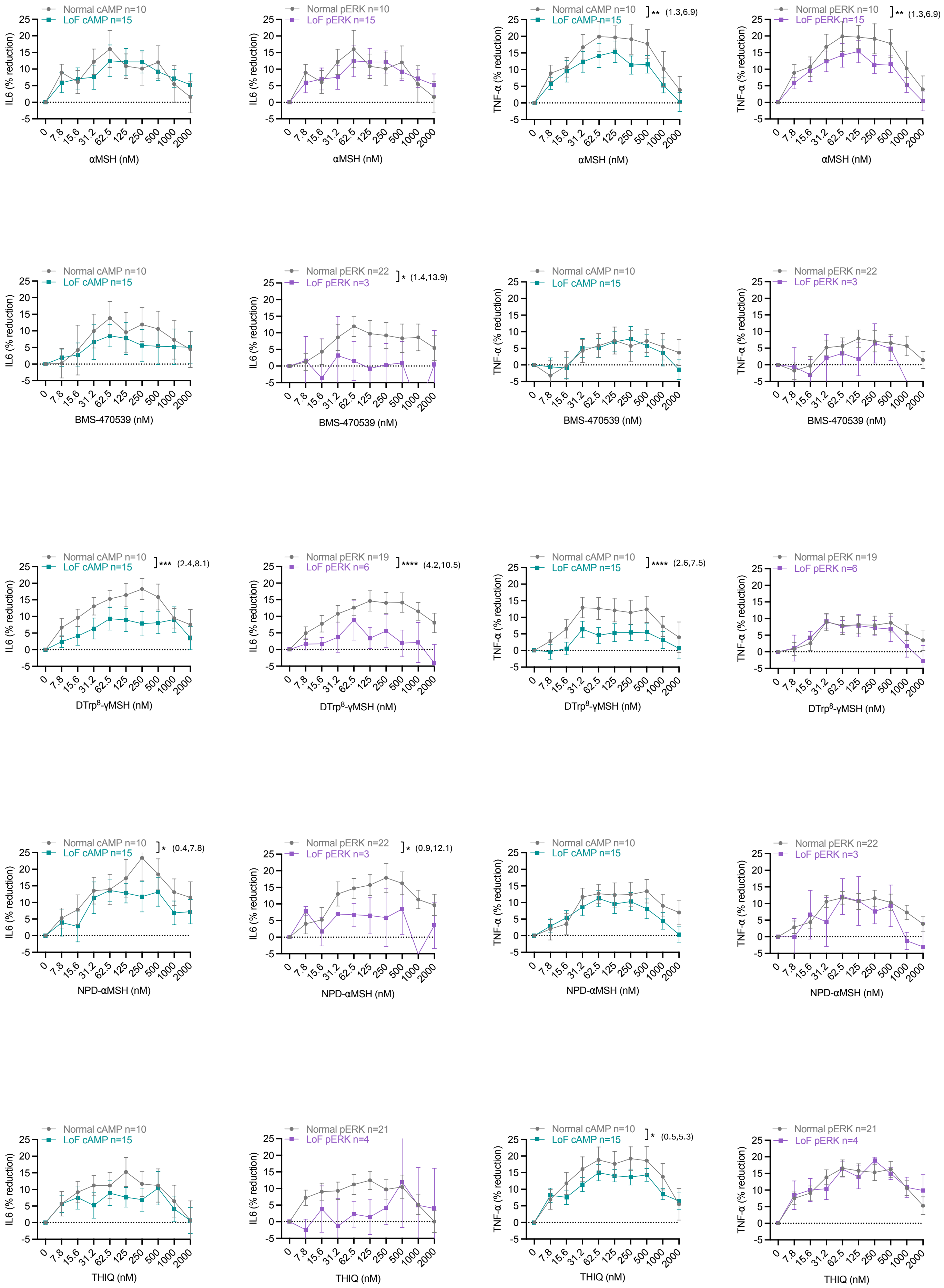

Normal cAMP n=10
LoF cAMP n=15
IL6 (% reduction)
αMSH (nM)
Normal pERK n=10
LoF pERK n=15
IL6 (% reduction)
αMSH (nM)
Normal cAMP n=10
LoF cAMP n=15
]** (1.3,6.9)
TNF-α (% reduction)
αMSH (nM)
Normal pERK n=10
LoF pERK n=15
]** (1.3,6.9)
TNF-α (% reduction)
αMSH (nM)
Normal cAMP n=10
LoF cAMP n=15
IL6 (% reduction)
BMS-470539 (nM)
Normal pERK n=22
LoF pERK n=3
]* (1.4,13.9)
IL6 (% reduction)
BMS-470539 (nM)
Normal cAMP n=10
LoF cAMP n=15
TNF-α (% reduction)
BMS-470539 (nM)
Normal pERK n=22
LoF pERK n=3
TNF-α (% reduction)
BMS-470539 (nM)
Normal cAMP n=10
LoF cAMP n=15
]*** (2.4,8.1)
IL6 (% reduction)
DTrp⁸-γMSH (nM)
Normal pERK n=19
LoF pERK n=6
]**** (4.2,10.5)
IL6 (% reduction)
DTrp⁸-γMSH (nM)
Normal cAMP n=10
LoF cAMP n=15
]**** (2.6,7.5)
TNF-α (% reduction)
DTrp⁸-γMSH (nM)
Normal pERK n=19
LoF pERK n=6
TNF-α (% reduction)
DTrp⁸-γMSH (nM)
Normal cAMP n=10
LoF cAMP n=15
]* (0.4,7.8)
IL6 (% reduction)
NPD-αMSH (nM)
Normal pERK n=22
LoF pERK n=3
]* (0.9,12.1)
IL6 (% reduction)
NPD-αMSH (nM)
Normal cAMP n=10
LoF cAMP n=15
TNF-α (% reduction)
NPD-αMSH (nM)
Normal pERK n=22
LoF pERK n=3
TNF-α (% reduction)
NPD-αMSH (nM)
Normal cAMP n=10
LoF cAMP n=15
IL6 (% reduction)
THIQ (nM)
Normal pERK n=21
LoF pERK n=4
IL6 (% reduction)
THIQ (nM)
Normal cAMP n=10
LoF cAMP n=15
]* (0.5,5.3)
TNF-α (% reduction)
THIQ (nM)
Normal pERK n=21
LoF pERK n=4
TNF-α (% reduction)
THIQ (nM)
25 20 15 10 5 0 -5
0 7.8 15.6 31.2 62.5 125 250 500 1000 2000

Figure S12. Effect of *MC1R* variants and cytokine responses in the whole-blood assay for melanocortin compounds. Concentration-response graphs for IL6 and for TNF-α displaying the response based on donors which are WT or have a functional variant (grey line) or whether they carry a LoF variant on cAMP (teal line) or a LoF variant on pERK (purple line) based on signalling data for each of the compounds. Data represent mean±SEM (n indicated in each graph) and was analysed by two-way ANOVA, *$p<0.05$, ***$p<0.001$, ****$p<0.0001$. 95% confidence intervals are provided in brackets.

Fig. S13

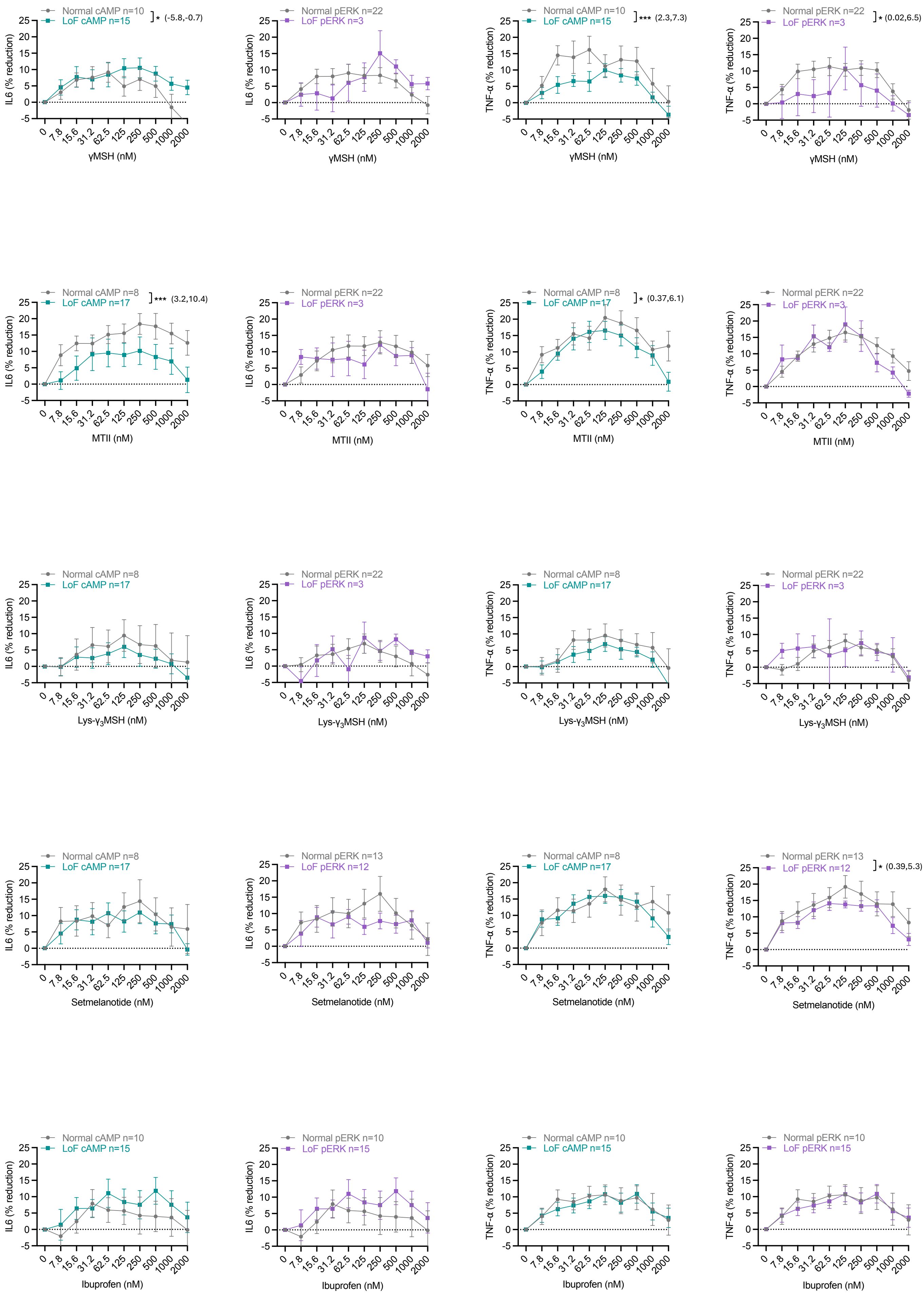

Normal cAMP n=10
LoF cAMP n=15
* (-5.8,-0.7)
IL6 (% reduction)
γMSH (nM)
Normal pERK n=22
LoF pERK n=3
IL6 (% reduction)
γMSH (nM)
Normal cAMP n=10
LoF cAMP n=15
*** (2.3,7.3)
TNF-α (% reduction)
γMSH (nM)
Normal pERK n=22
LoF pERK n=3
* (0.02,6.5)
TNF-α (% reduction)
γMSH (nM)
Normal cAMP n=8
LoF cAMP n=17
*** (3.2,10.4)
IL6 (% reduction)
MTII (nM)
Normal pERK n=22
LoF pERK n=3
IL6 (% reduction)
MTII (nM)
Normal cAMP n=8
LoF cAMP n=17
* (0.37,6.1)
TNF-α (% reduction)
MTII (nM)
Normal pERK n=22
LoF pERK n=3
TNF-α (% reduction)
MTII (nM)
Normal cAMP n=8
LoF cAMP n=17
IL6 (% reduction)
Lys-γ3MSH (nM)
Normal pERK n=22
LoF pERK n=3
IL6 (% reduction)
Lys-γ3MSH (nM)
Normal cAMP n=8
LoF cAMP n=17
TNF-α (% reduction)
Lys-γ3MSH (nM)
Normal pERK n=22
LoF pERK n=3
TNF-α (% reduction)
Lys-γ3MSH (nM)
Normal cAMP n=8
LoF cAMP n=17
IL6 (% reduction)
Setmelanotide (nM)
Normal pERK n=13
LoF pERK n=12
IL6 (% reduction)
Setmelanotide (nM)
Normal cAMP n=8
LoF cAMP n=17
TNF-α (% reduction)
Setmelanotide (nM)
Normal pERK n=13
LoF pERK n=12
* (0.39,5.3)
TNF-α (% reduction)
Setmelanotide (nM)
Normal cAMP n=10
LoF cAMP n=15
IL6 (% reduction)
Ibuprofen (nM)
Normal pERK n=10
LoF pERK n=15
IL6 (% reduction)
Ibuprofen (nM)
Normal cAMP n=10
LoF cAMP n=15
TNF-α (% reduction)
Ibuprofen (nM)
Normal pERK n=10
LoF pERK n=15
TNF-α (% reduction)
Ibuprofen (nM)
0 7.8 15.6 31.2 62.5 125 250 500 1000 2000
-5 0 5 10 15 20 25

Figure S13. Effect of *MC1R* variants and cytokine responses in the whole-blood assay for melanocortin compounds and positive controls. Concentration-response graphs for IL6 and for TNF-α displaying the response based on donors which are WT or have a functional variant (grey line) or whether they carry a LoF variant on cAMP (teal line) or a LoF variant on pERK (purple line) based on signalling data for each of the compounds. For ibuprofen, LoF refers to signalling data for αMSH. Data represent mean±SEM (n indicated in each graph)and was analysed by two-way ANOVA, *p<0.05, ***p<0.001, ****p<0.0001. 95% confidence intervals are provided in brackets.

Fig. S14

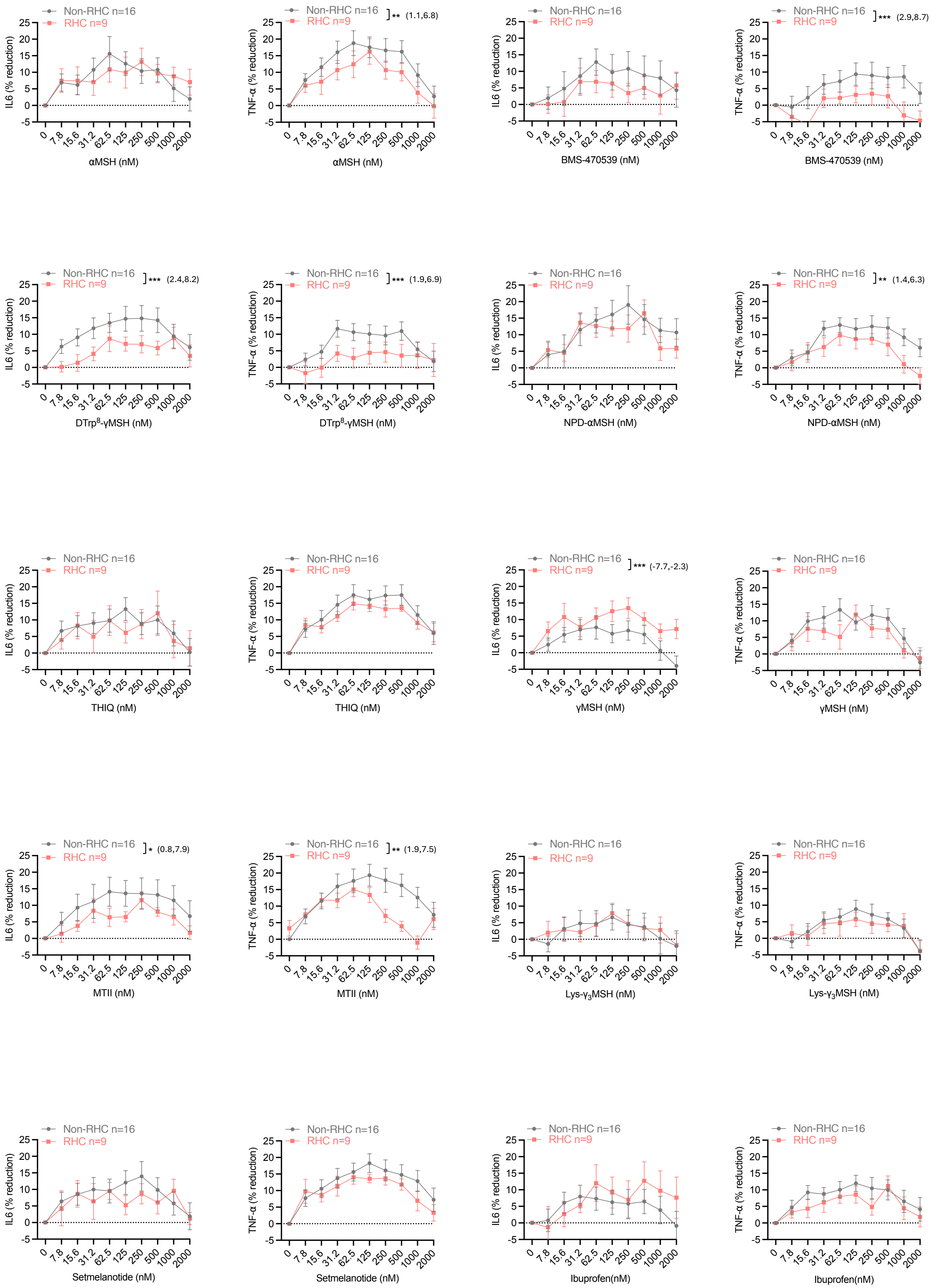
Non-RHC n=16
RHC n=9
IL6 (% reduction)
TNF-α (% reduction)
αMSH (nM)
BMS-470539 (nM)
DTrp8-γMSH (nM)
NPD-αMSH (nM)
THIQ (nM)
γMSH (nM)
MTII (nM)
Lys-γ3MSH (nM)
Setmelanotide (nM)
Ibuprofen(nM)
** (1.1,6.8)
*** (2.9,8.7)
*** (2.4,8.2)
*** (1.9,6.9)
** (1.4,6.3)
*** (-7.7,-2.3)
* (0.8,7.9)
** (1.9,7.5)
0
7.8
15.6
31.2
62.5
125
250
500
1000
2000
25
20
15
10
5
0
-5

Figure S14. Effect of RHC variants on *MC1R* and cytokine responses in the whole-blood assay for melanocortin compounds and positive controls. Concentration-response graphs for IL6 and for TNF-α displaying the response based on donors which are WT or have a functional variant (grey line) or whether they carry a variant described as being associated with red hair, RHC (red line). Data represent mean±SEM (n indicated in each graph) and was analysed by two-way ANOVA, *$p<0.05$, **$p<0.01$, ***$p<0.001$. 95% confidence intervals are provided in brackets.

Fig. S15

A

Characteristic		Mean/units	SD
Age	Years	30.92	8.976
Female	n (%)	11 (44)	
Skin phototype	Type (I-VI)	2.88	1.201
Fitzpatrick score	score (max 40)	16.92	7.511
Genetic	score (max 16)	8.16	3.944
Sensitivity	score (max 16)	6.84	3.727
Intentional exposure	score (max 8)	1.92	1.754
Ethnicity			
White	n (%)	15 (60)	
Black	n (%)	1 (4)	
East Asia	n (%)	2 (8)	
South Asia	n (%)	4 (16)	
Latino	n (%)	1 (4)	
Mixed	n (%)	2 (8)	

B

	Score				
**Fitzpatrick Questionnaire**	**0**	**1**	**2**	**3**	**4**
**Genetic (physical traits)**					
What are the colour of your eyes?	Light blue, green, grey	Blue, green, grey	Dark blue, green, light brown	Dark brown	Brownish black
What is the colour of your hair (naturally and before aging)?	Red	Blonde	Chestnut or dark blonde	Dark brown	Black
What is the colour of your skin (unexposed areas)?	Pink	Very pale	Light brown or olive	Brown	Dark brown
Do you have freckles on unexposed areas?	Many	Several	Few	Rare	None
**Sensitivity (reaction to sun exposure)**					
What happens to you skin if you stay in the sun for an extended period?	Severe burns, blistering peeling	Moderate burns, blistering, peeling	Burns sometimes, followed by peeling	Rare burns	No burns
Do you turn brown after sun exposure?	Never	Rarely	Sometimes	Often	Always
How brown do you get?	Hardly or not at al	Light tan	Medium tan	Dark tan	Very dark tan
Is your face sensitive to the sun?	Very sensitive	Sensitive	Mildly sensitive	Resistant	Very resistant
**Intentional exposure (tanning habits)**					
How often to you tan?	Never	Rarely	Sometimes	Often	Always
When did you last expose your skin to the sun or artificial tanning sources?	>3 months ago	Last 2-3 months	Last 1-2 months	Last week	Last day

**Fitzpatrick score:** Genetic + Sensitivity + Intentional (max 40)	**Skin Phototype**
0-6	I
7-13	II
14-20	III
21-27	IV
28-34	V
35+	VI

Figure S15. Demographic characteristics and skin parameters for the healthy blood donors. **A)** Mean or units (female and ethnicity), and standard deviation (SD) are shown. Skin phototype, Fitzpatrick score, genetic, sensitivity and intentional exposure parameters are based on the donor's responses to the Fitzpatrick questionnaire shown in **B**.

Fig. S16

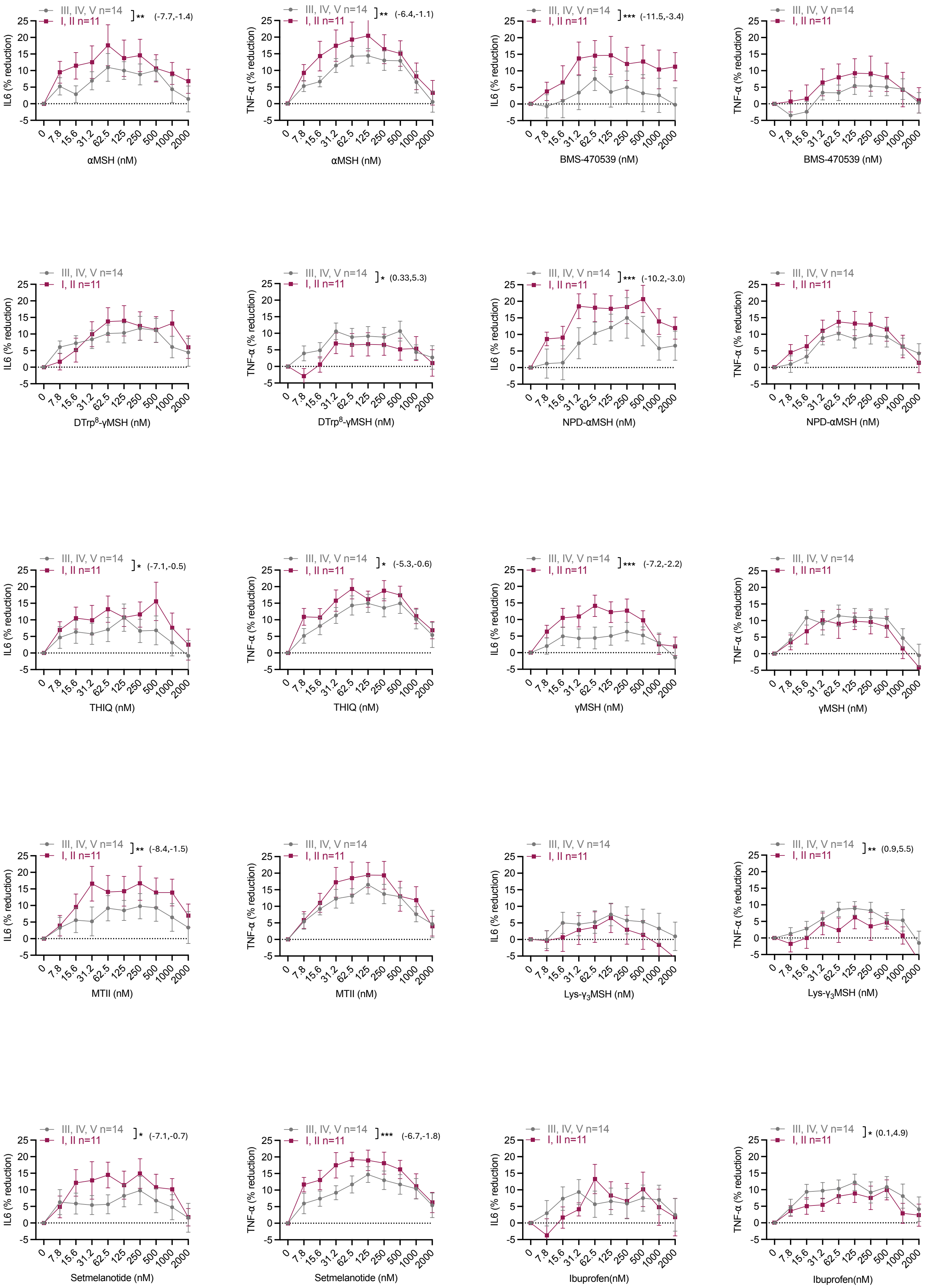

III, IV, V n=14
I, II n=11
** (-7.7,-1.4)
IL6 (% reduction)
αMSH (nM)
** (-6.4,-1.1)
TNF-α (% reduction)
αMSH (nM)
*** (-11.5,-3.4)
IL6 (% reduction)
BMS-470539 (nM)
TNF-α (% reduction)
BMS-470539 (nM)
IL6 (% reduction)
DTrp^{8}-γMSH (nM)
* (0.33,5.3)
TNF-α (% reduction)
DTrp^{8}-γMSH (nM)
*** (-10.2,-3.0)
IL6 (% reduction)
NPD-αMSH (nM)
TNF-α (% reduction)
NPD-αMSH (nM)
* (-7.1,-0.5)
IL6 (% reduction)
THIQ (nM)
* (-5.3,-0.6)
TNF-α (% reduction)
THIQ (nM)
*** (-7.2,-2.2)
IL6 (% reduction)
γMSH (nM)
TNF-α (% reduction)
γMSH (nM)
** (-8.4,-1.5)
IL6 (% reduction)
MTII (nM)
TNF-α (% reduction)
MTII (nM)
IL6 (% reduction)
Lys-γ$_{3}$MSH (nM)
** (0.9,5.5)
TNF-α (% reduction)
Lys-γ$_{3}$MSH (nM)
* (-7.1,-0.7)
IL6 (% reduction)
Setmelanotide (nM)
*** (-6.7,-1.8)
TNF-α (% reduction)
Setmelanotide (nM)
IL6 (% reduction)
Ibuprofen(nM)
* (0.1,4.9)
TNF-α (% reduction)
Ibuprofen(nM)
25 20 15 10 5 0 -5
0 7.8 15.6 31.2 62.5 125 250 500 1000 2000

Figure S16. Effect of skin phototype and cytokine responses in the whole-blood assay for melanocortin compounds . A) Concentration-response graphs for IL6 and for TNF-α displaying the response based on donors with skin phototypes III, IV or V (grey line) or skin phototypes I or II (plum line). Data represent mean±SEM (n indicated in the graphs)and was analysed by two-way ANOVA, *$p<0.05$, **$p<0.01$, ***$p<0.001$. 95% confidence intervals are provided in brackets.
